# A protein-based pneumococcal vaccine elicits broad immunity associated with multifunctional antibody responses in humans

**DOI:** 10.1172/JCI196261

**Published:** 2026-02-02

**Authors:** Kaiyi Li, Jinglu Yang, Xiaobing Zhai, Jinbo Gou, Xiuwen Sui, Bochao Wei, Yuan Wang, Xiaoling Su, Xiaoyun Yang, Shiqin Jin, Xuan Zhou, Yuxuan Zhang, Tao Zhu, Junxiang Wang, Zhongfang Wang

**Affiliations:** 1 State Key Laboratory of Respiratory Disease, National Clinical Research Center for Respiratory Disease, Guangzhou Institute of Respiratory Health, The First Affiliated Hospital of Guangzhou Medical University, Guangzhou, China.; 2 Guangzhou National Laboratory, Guangzhou, China.; 3 CanSino Biologics Inc., Tianjin, China.; 4 Key Laboratory of Emergency and Trauma of Ministry of Education, Engineering Research Center for Hainan Biological Sample Resources of Major Diseases, The Hainan Branch of National Clinical Research Center for Cancer, The First Clinical College, The First Affiliated Hospital, Hainan Medical University, Haikou, China.; 5 Shenzhen Hetao Institute of Guangzhou National Laboratory, Guangzhou, China.

**Keywords:** Immunology, Public Health, Bacterial vaccines

## Abstract

Traditional polysaccharide vaccines are constrained by streptococcus pneumoniae diversity. We propose a protein-based pneumococcal vaccine (PBPV) — formulated with conserved surface proteins P3296, P5668, PRx1, and pneumolysin (Ply) — that could potentially offer superior immune breadth independent of capsular polysaccharide serotypes. Here, we evaluated the multifunctional antibody responses induced by PBPV, including immunogenicity, Ply neutralization, opsonophagocytic activity (OPA), and such nonopsonic functions as NK cell activation (ADNKA), antibody-dependent cellular phagocytosis, and neutrophil phagocytosis (ADNP) in a cohort of 50- to 69-year-olds. While PBPV showed shorter-lasting immune responses, including reduced Ply-neutralizing capacity, it provided broader cross-serotype protection than 23-valent pneumococcal polysaccharide vaccine. Correlation analysis identified distinct PspA-specific IgG subclass roles: P3296-IgG1 correlated with OPA, and IgG3 correlated with ADNKA/ADNP; P5668-IgG2 correlated with ADNKA/ADNP, and IgG3 correlated with OPA; and PRx1-IgG2 correlated with OPA, and IgG3 correlated with ADNKA. Critically, while no efficacy data have yet confirmed the protective effect of PBPV, its targeting of conserved proteins rather than capsular polysaccharides enables simplified manufacturing and expanded coverage, positioning it as a promising alternative to traditional multipolysaccharide vaccines.

## Abstract 2

PBPV elicits multifunctional antibodies (opsonophagocytosis, antibody-dependent cellular activation and phagocytosis) through conserved pneumococcal protein targets, enabling broader cross-serotype protection and streamlined manufacturing compared to traditional polysaccharide vaccines.

## Graphical abstract

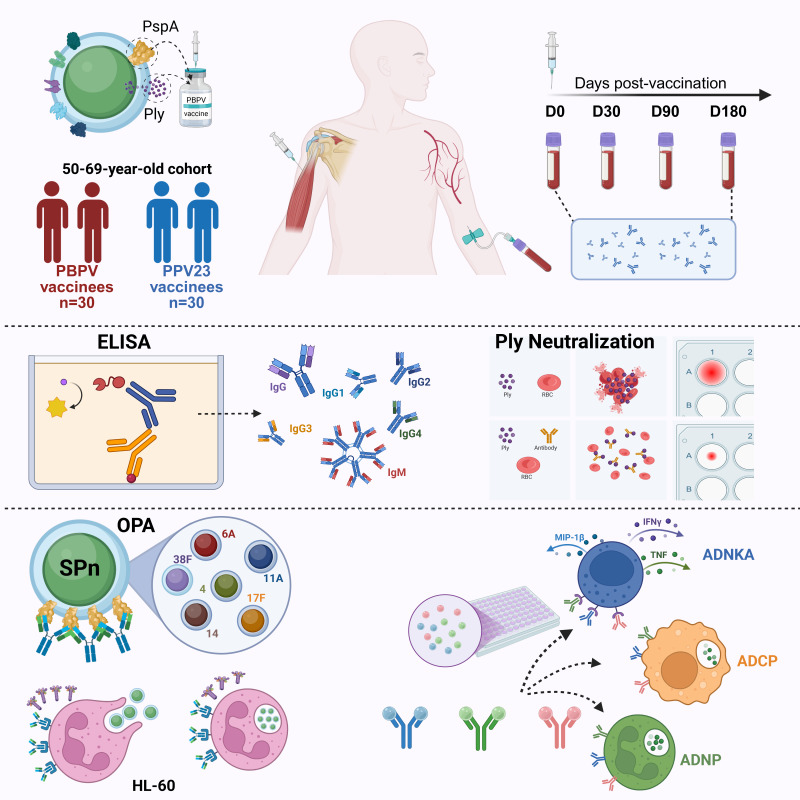

## Introduction

Streptococcus pneumoniae (SPn), a prominent respiratory pathogen, continues to pose substantial global health challenges through its association with invasive pneumococcal diseases (IPDs) such as bacterial pneumonia (accounting for 40%–50% of community-acquired pneumonia cases), otitis media, meningitis, and septicemia (1, 2). Recent epidemiological surveillance data revealed that adults ≥ 70 years old experience 3–5 times higher IPD incidence rates compared with younger populations, with case fatality rates exceeding 20% in geriatric patients with comorbidities (3). This vulnerability is exacerbated by antibiotic resistance, as demonstrated by 2024 surveillance data showing resistance to erythromycin and tetracyclines resistance in more than 83% of Chinese isolates and emerging resistance to trimethoprim/sulfamethoxazole in more than 50% of Chinese isolates (4). The antimicrobial resistance (AMR) crisis has dire consequences: modeling predicts 39 million AMR-related deaths globally between 2025 and 2050, with SPn contributing substantially to respiratory infection mortality (5, 6). Notably, age-stratified analysis indicates an 83% increase in AMR-attributable mortality among septuagenarians since 2020 (5). These data underscore the urgent need for enhanced immunization strategies and antimicrobial stewardship programs targeting vulnerable geriatric populations.

While SPn vaccination has reduced disease burden, with pneumococcal conjugate vaccines (PCVs) decreasing IPD incidence by 72%–83%, current vaccines — 23-valent pneumococcal polysaccharide vaccine (PPV23) and PCV13 — face marked limitations (7, 8). They protect against only 60%–70% of circulating strains, and nonvaccine types now account for 40% of IPD cases in adults aged ≥65 years (9). Implementing polysaccharide-based vaccines is hindered by manufacturing complexity, antigenic variation, and reduced immunogenicity. Protein-based vaccines, targeting conserved pneumococcal proteins, have emerged as a promising solution, demonstrating good efficacy against multiple serotypes in clinical trials (10, 11). These vaccines induce both humoral and cellular immunity, providing more durable protection, particularly in the elderly, while mitigating serotype replacement risks (12).

In addition to capsular polysaccharides, surface-exposed virulence proteins of SPn, particularly pneumococcal surface protein A (PspA) and pneumolysin (Ply), have emerged as promising immunogens for next-generation vaccine development (13). PspA, a choline-binding protein, modulates host immune responses by inhibiting complement deposition and opsonophagocytosis, while also conferring resistance to lactoferrin-mediated bacterial killing (14, 15). Ply, a pore-forming cytotoxin, disrupts ciliary function in the respiratory epithelium and enhances bacterial adherence to damaged bronchial cells, facilitating SPn invasion into the lower respiratory tract (16, 17). Preclinical studies have demonstrated that antibodies targeting PspA and Ply confer robust protection in murine models, with synergistic effects observed when these proteins are coadministered (18). Although PspA exhibits sequence variability in its N-terminal α-helical domain, leading to its classification into distinct clades, antibodies against PspA within the same clade exhibit cross-protective activity, and multiclade formulations can provide broader serotype coverage compared with polysaccharide-based vaccines (19–21). Ply, in contrast, is highly conserved across all SPn serotypes, making it an ideal candidate for universal vaccine development (22). This multiprotein strategy enhances immune protection breadth and durability, addressing polysaccharide vaccine limitations such as serotype replacement and incomplete coverage (23).

Building on the development of a recombinant protein-based pneumococcal vaccine (PBPV) by CanSino Biologics Inc. (24), which incorporates PspA-3296 (clade 3), PspA-5668 (clade 4), PspA-PRx1 (clade 2), and Ply as immunogens, we conducted a comprehensive evaluation of its immunogenicity, antibody profile composition, and functional antibody responses in a cohort of 50- to 69-year-olds (Tables 1 and 2). This study provides a rigorous assessment of PBPV efficacy in this high-risk demographic, addressing critical gaps in pneumococcal vaccine development for aging populations. By elucidating the vaccine’s ability to elicit robust and functional immune responses, our findings offer robust theoretical and empirical support for the advancement and implementation of next-generation SPn vaccines, particularly those leveraging multiprotein formulations to overcome the limitations of traditional polysaccharide-based approaches.

## Results

### PBPV elicits greater humoral responses at day 30 after vaccination, but lower Ply neutralization, relative to the conventional polysaccharide-based vaccine.

**Figure 1:**
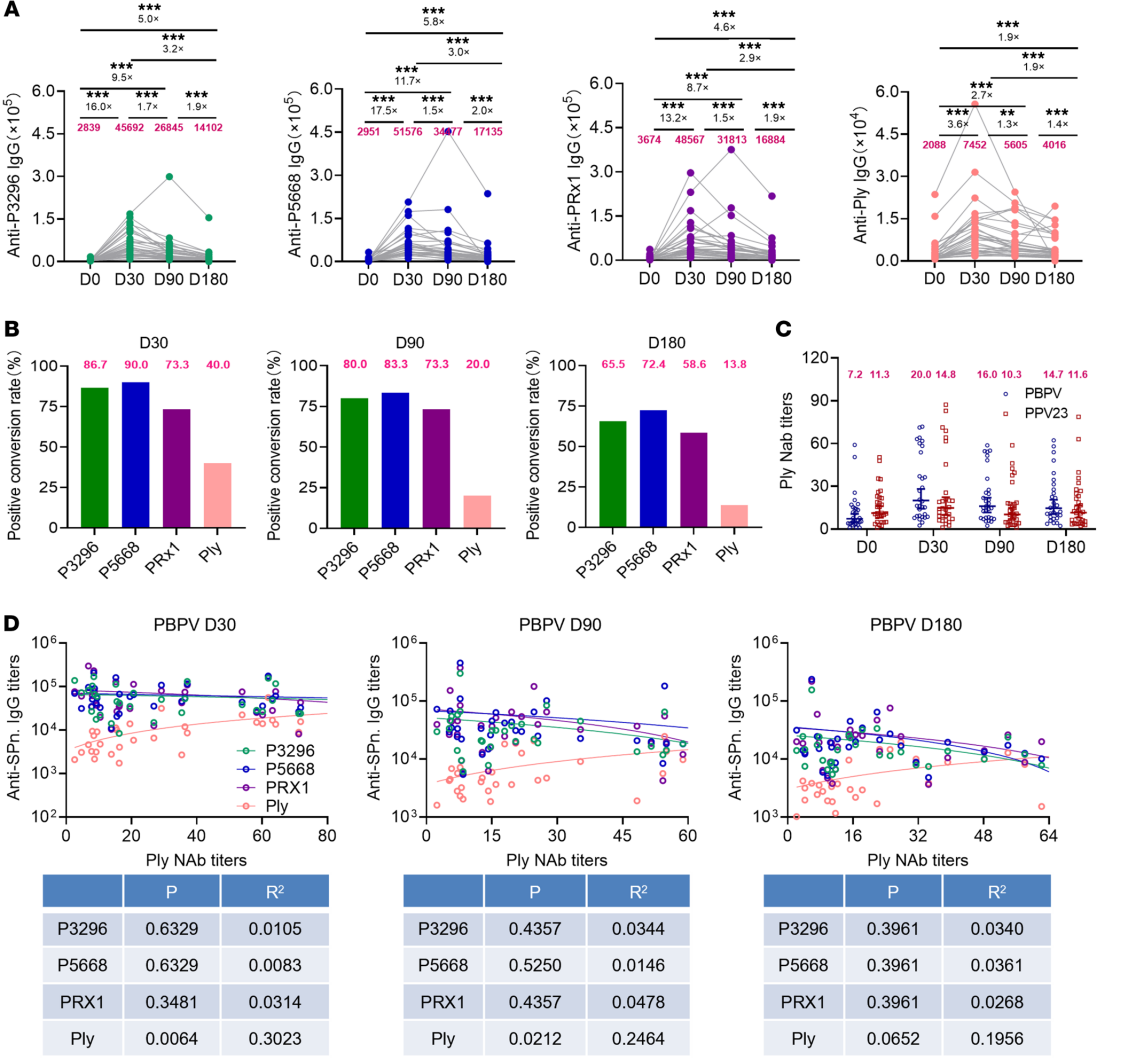
Binding antibodies and neutralizing antibodies induced by PBPV. (**A**) Specific antibody titers against P3296 (green), P5668 (blue), PRx1 (purple), and Ply (pink) were measured in the PBPV group (*n* = 30) at D0, D30, D90, and D180. (**B**) Positive conversion rates for P3296, P5668, PRx1, and Ply were assessed at D30, D90 and D180; positive response defined as a ≥4-fold increase in antibody titers compared with D0 levels. (**C**) Comparison of neutralizing antibody titers between the PBPV and PPV23 groups; data are shown as geometric mean with 95% CI. (**D**) Correlation analysis between specific antibody titers of the 4 PBPV immunogens (P3296, P5668, PRx1, and Ply) and neutralizing antibody titers against Ply. The numbers in magenta indicate the geometric mean titers in **A** and **C** and positive conversion rates in **B**. Significance was measured using Wilcoxon’s rank-sum test in **A**, Mann-Whitney test in **C**, and simple linear regression analysis in **D**. All *P* values were adjusted for multiple testing using the Benjamini–Hochberg (BH) method. **P < 0.01, ***P < 0.001.

To assess the immunogenicity of PBPV, we measured specific IgG antibody titers against P3296, P5668, PRx1, and Ply in the serum of 30 vaccinees at days 0, 30, 90, and 180 after vaccination (D0, D30, D90, and D180) (Figure 1A). The results demonstrated that PBPV effectively induced antibodies against all 4 proteins, with titers peaking at D30 and gradually declining thereafter. At D30, antibody titers against P3296, P5668, PRx1, and Ply were 16.0-, 17.5-, 13.2-, and 3.6-fold higher, respectively, compared with baseline levels at D0. Analysis of positive conversion rates (fold change ≥ 4) revealed that PspA-specific antibodies (P3296, P5668, and PRx1) exhibited markedly higher rates than Ply-specific antibodies. At D30, the positive conversion rates were 86.7% for P3296, 90.0% for P5668, 73.3% for PRx1, and only 40.0% for Ply. Notably, P5668 showed the highest positive conversion rate across all time points, while PRx1 maintained a stable rate between D30 and D90. In contrast, the positive conversion rate for Ply was consistently lower than that of PspA-specific antibodies and declined more rapidly (Figure 1B and Supplemental Figure 1A; supplemental material available online with this article; https://doi.org/10.1172/JCI196261DS1). In addition, we also measured the changes in antibody titers against P3296, P5668, PRx1, and Ply in individuals vaccinated with PPV23 at D0–D180. Although vaccination with PPV23 only slightly increased antibody titers at D30, it barely induced effective positive conversion rates (Supplemental Figure 2, A and B). These results highlight the superior immunogenicity of PspA-specific antibodies induced by PBPV, with sustained responses lasting up to D180, whereas Ply-specific antibodies showed weaker induction and maintenance.

Given the conserved nature and host cell–lysing properties of Ply, we further investigated the neutralizing ability of Ply-specific antibodies induced by PBPV. Using the rabbit erythrocyte-Ply lysis inhibition assay, we measured Ply-neutralizing antibody titers in the PBPV and PPV23 groups at D0, D30, D90, and D180 (Figure 1C). Although the PBPV group reached peak neutralizing antibody levels at D30, the increase was modest compared with baseline levels. Moreover, the Ply neutralization capacity of the PBPV group was comparable with that of the PPV23 group. Correlation analysis revealed that only Ply-specific antibody titers were significantly associated with Ply neutralization titers (Figure 1D). However, the Ply-neutralizing capacity of individuals vaccinated with PPV23 was largely derived from preexisting Ply-neutralizing antibodies and was not associated with the duration of PPV23 vaccination (Supplemental Figure 3, A–E). These results suggest that while PBPV induces Ply-specific antibodies, its ability to generate Ply-neutralizing antibodies remains limited.

### PBPV induces a broader but less sustained opsonophagocytic ability compared with PPV23.

Building on the findings that PBPV effectively induces PspA- and Ply-specific antibodies, with superior immunogenicity for PspA-specific responses but limited Ply-neutralizing activity, we further investigated whether these antibodies could mediate functional immune protection. To evaluate this, we performed opsonophagocytic activity (OPA) assays, which simulate in vivo immune protection by measuring antibody-mediated opsonophagocytic killing of bacteria in the presence of complement. Additionally, to assess whether PBPV provides broader immune protection against different SPn serotypes and to compare its immune protection strength and maintenance with the traditional PPV23, we measured the OPA indices in both PBPV- and PPV23-vaccinated populations. The OPA assay included 4 SPn serotypes (4, 11A, 17F, and 14) covered by PPV23 and 2 serotypes (6A and 38F) not included in PPV23, representing the 3 PspA families. Measurements were taken at D0, D30, D90, and D180.

**Figure 2:**
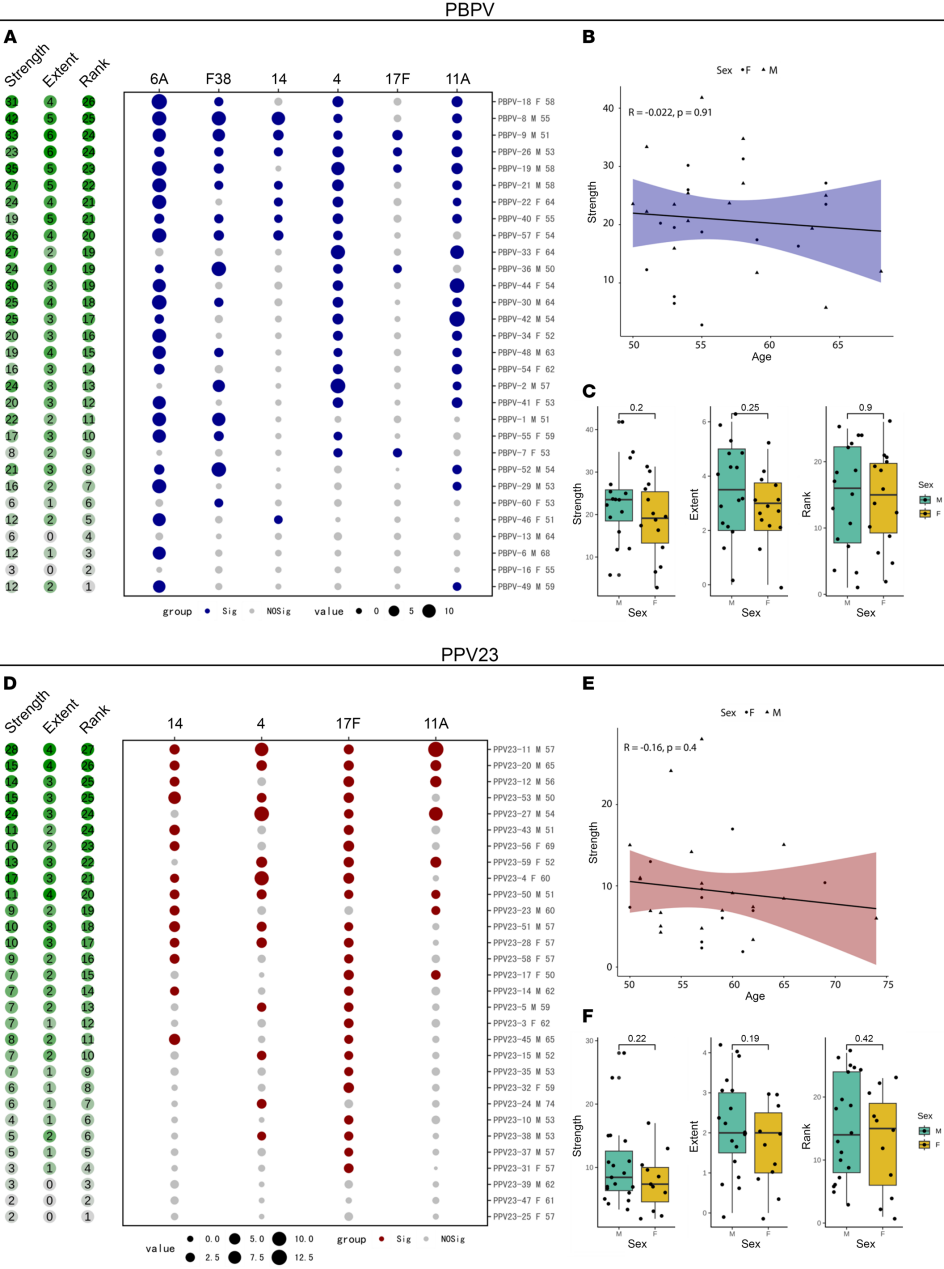
Comparative analysis of OPA induced by PBPV and PPV23 at D30. (**A** and **D**) The OPA elicited by PBPV and PPV23 at D30 is presented. The left panel illustrates the strength [log_2_(sum fold change)], extent, and rank of OPA reactions: strength means the sum of fold change values between baseline and D30 for all serotypes; extent means the number of serotypes (of 4) to which the donor elicits marked (that is, an OPA titer of ≥2) responses; and rank means an individual’s vaccine responsiveness in the cohort based on aggregate responses for all serotypes, where higher ranks represent stronger responses. The bubble plot uses color coding to indicate marked responses (log_2_ fold change > 1): blue and red denote a marked enhancement of OPA functionality (D30/D0 OPA value ≥ 2), and gray indicates a value ≤ 2. Bubble size represents the magnitude of the response. Information on participant ID, gender (F for female, M for male), and age is provided at right. (**B** and **E**) Correlation between OPA response intensity and age at D30 with PBPV and PPV23. (**C** and **F**) Gender-based differences in OPA response intensity, magnitude, and grade at D30 with PBPV and PPV23. Box-and-whisker plots display the median and IQR (25%–75%), with whiskers indicating values within 1.5 × IQR above and below the quartiles.

The results revealed distinct serotype-specific immune responses between the PBPV and PPV23 groups. The PBPV group exhibited strong OPA against serotypes 6A, 4, and 11A, while the PPV23 group showed a dominant response primarily against serotype 17F (Figure 2, A and D). This indicates that PBPV induces broader immune protection across multiple serotypes, whereas PPV23 elicits a more targeted response to specific serotypes. Further analysis of the OPA indices for 4 pneumococcal serotypes (4, 14, 11A, and 17F) included in PPV23 showed that at least 7 individuals (7/30) in the PBPV group and 10 individuals (10/30) in the PPV23 group demonstrated OPA against at least 3 serotypes (Figure 2, A and D). Although the dominant serotype responses in both groups remained consistent at D90 and D180, the strength of the responses declined over time (Supplemental Figure 4, A and D, and Supplemental Figure 5, A and D). Notably, no significant correlation was observed between response strength and age in either the PBPV or PPV23 group (Figure 2, B and E, Supplemental Figure 4, B and E, and Supplemental Figure 5, B and E), which contrasts with the previously reported age-dependent effectiveness of PCV13 (25). Similarly, when comparing gender among vaccine recipients, no significant differences were observed in response strength, extent, or rank (Figure 2, C and F, Supplemental Figure 4, C and F, and Supplemental Figure 5, C and F).

**Figure 3:**
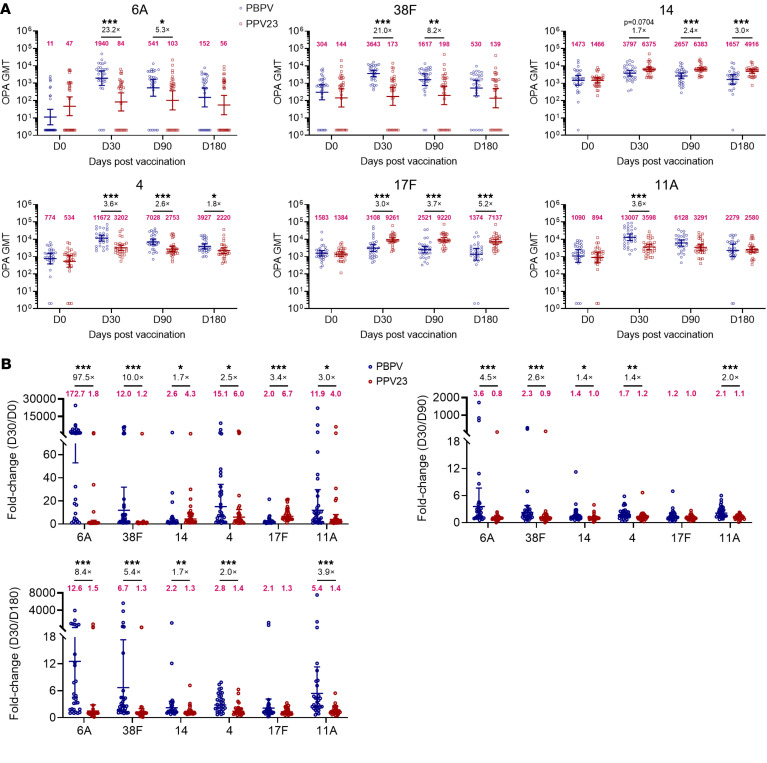
Comparative analysis of OPA between the PBPV and PPV23 groups. (**A**) OPA mediated by 6 serotypes (6A, 38F, 4, 11A, 14, and 17F) was assessed in 60 vaccine recipients (PBPV group, *n* = 30; PPV23 group, *n* = 30) at D0, D30, D90, and D180. The differences in OPA between the PBPV and PPV23 groups were evaluated across these time points. (**B**) Fold changes in OPA indices were analyzed to compare differences between PBPV and PPV23 at D30/D0, D30/D90, and D30/D180. The numbers in magenta indicate the geometric mean titers (GMT) in **A** and the mean fold change in each group in **B**. Significance was measured using the Mann-Whitney test in **A** and **B**. Data are shown as geometric mean with 95% CI in **A** and **B**. All *P* values were adjusted for multiple testing using the BH method. *P < 0.05, **P < 0.01, ***P < 0.001.

To further characterize the distinct immune protection profiles of PBPV and PPV23, we compared their opsonophagocytic activities against different SPn serotypes. PBPV demonstrated broad-spectrum efficacy, successfully mediating opsonophagocytosis against 2 serotypes (6A and 38F) not covered by PPV23, with activity persisting until D90 (Figure 3A). For serotypes 4, 14, 11A, and 17F, which are included in PPV23, both vaccines induced varying levels of OPA from D30 to D180. Notably, PPV23 exhibited superior activity against serotype 14 (D30: 6,375 vs. 3,797, 1.7-fold; D90: 6,383 vs. 2,657, 2.4-fold; D180: 4,916 vs. 1,657, 3.0-fold) and serotype 17F (D30: 9,261 vs. 3,108, 3.0-fold; D90: 9,220 vs. 2,521, 3.7-fold; D180: 7,137 vs. 1,374, 5.2-fold) compared with PBPV. In contrast, PBPV induced stronger activity against serotype 4 (D30: 11,672 vs. 3,202, 3.6-fold; D90: 7,028 vs. 2,753, 2.6-fold; D180: 3,927 vs. 2.220, 1.8-fold) and serotype 11A (D30: 13,007 vs. 3,598, 3.6-fold; D90: 6,128 vs. 3,291, 1.9-fold) compared with PPV23 (Figure 3A).

It is noteworthy that the temporal kinetics of immune protection revealed critical differences. While PBPV achieved rapid OPA induction (D30/D0 ratios exceeding PPV23), its activity declined more sharply over time, as evidenced by steeper D30/D180 ratios (Figure 3B). This contrasted with PPV23’s comparatively stable maintenance of OPA levels, particularly for its dominant serotypes. These findings clearly showed that PBPV is a broader candidate capable of targeting non-PPV23 serotypes, albeit with shorter-lived efficacy, while PPV23 maintains superiority for specific high-risk serotypes through durable protection.

### PRx1-specific IgG3 exhibits weaker induction potency but stronger persistence.

**Figure 4:**
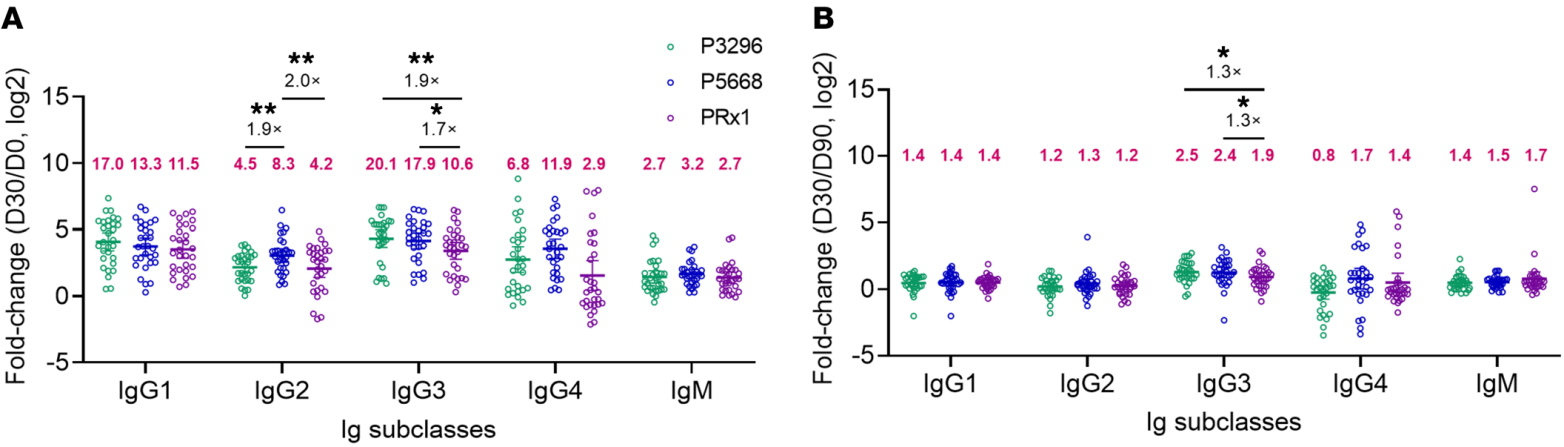
Antibody subclasses induced by PBPV. (**A** and **B**) Fold change in titers of antibody subclasses (IgG1, IgG2, IgG3, IgG4, and IgM) induced against P3296 (green), P5668 (blue), and PRx1 (purple) in vaccinated subjects. The numbers in magenta indicate the mean fold change in each group. Significance was measured using a paired, 2-tailed *t* test or Wilcoxon’s rank-sum test in **A** and **B**. Data are shown as geometric mean with 95% CI. All *P* values were adjusted for multiple testing using the BH method. *P < 0.05, **P < 0.01.

Characterizing the antibody subclass profiles is essential for understanding the composition and functional properties of the antibody repertoire induced by PBPV. The distinct composition of antibody subclasses, including IgG1, IgG2, IgG3, and IgG4, plays a critical role in determining the functional properties of antibodies, such as complement activation, opsonophagocytosis, and Fc-mediated effector functions. We analyzed the levels of P3296-, P5668-, and PRx1-specific IgG1, IgG2, IgG3, and IgG4 subclasses at 4 time points using ELISA. The overall kinetic trends of IgG1, IgG2, and IgG3 for P3296, P5668, and PRx1 were consistent with those of total IgG. Notably, IgG4 antibody titers for all 3 proteins were sustained at D180 after peaking at D30, showing no marked decline (Supplemental Figure 6, A–C). We then compared the induction (D30/D0) and maintenance (D30/D90) capabilities of the 3 protein-specific antibody subclasses. In terms of induction ability, P5668-specific IgG2 levels were substantially higher than those of P3296 (8.3 vs. 4.5, 1.9-fold) and PRx1 (8.3 vs. 4.2, 2.0-fold). PRx1-specific IgG3 levels were lower than those of P3296 (10.6 vs. 20.1, 1.9-fold) and P5668 (10.6 vs. 17.9, 1.7-fold). Similarly, PRx1-specific IgG4 levels tended to be lower than those of P5668 (2.9 vs. 11.9, 4.0-fold) (Figure 4A). Regarding maintenance ability, PRx1-specific IgG3 levels were higher than those of P3296 (2.5 vs. 1.9, 1.3-fold) and P5668 (2.4 vs. 1.9, 1.3-fold) (Figure 4B). These results indicate that the induction ability of PRx1-specific antibody subclasses was generally weaker compared with P3296 and P5668, while all 3 proteins exhibited similar maintenance abilities overall.

### PspA-specific antibody subclasses mediating OPA differ across post-PBPV immunization time points.

**Figure 5:**
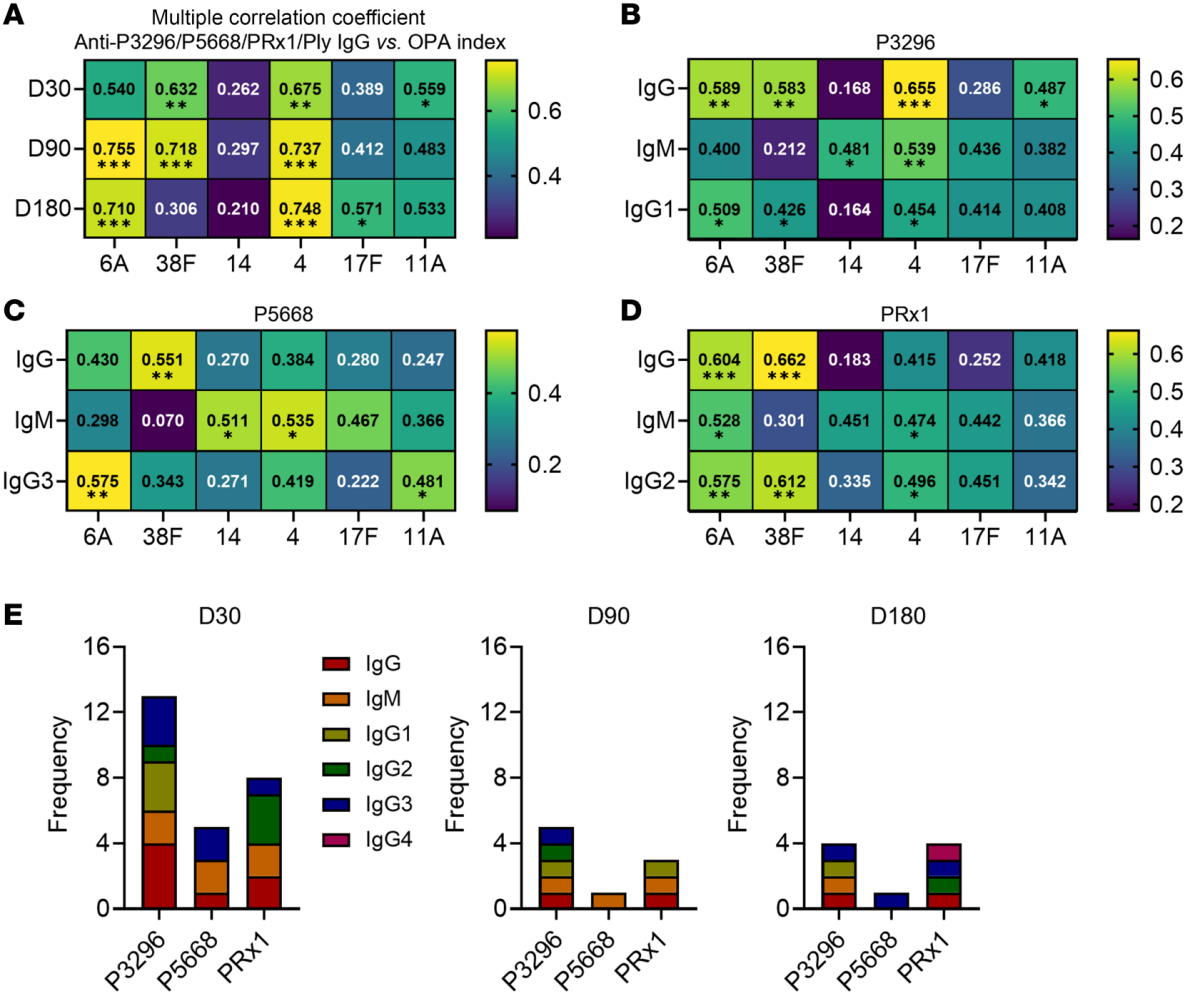
The dominant antibody subclass correlating OPA with different serotypes varies among different PspA proteins. (**A**) Multiple correlation coefficient analysis was conducted between the OPA indices for 6 serotypes (6A, 38F, 14, 4, 17F, and 11A) and the composite antibody titers of 4 immunogens in 30 subjects at D30, D90, and D180. The heatmap presents the correlation coefficients and corresponding *P* values. (**B**–**D**) Spearman’s correlation analysis between the antibody subclasses of P3296, P5668, and PRx1 and the OPA indices for the 6 serotypes at D30. (**E**) The frequency of serotype-specific responses elicited by different antibody subclasses at D30, D90, and D180. Significance was measured using Spearman’s correlation analysis in **A**–**D**. All *P* values were adjusted for multiple testing using the BH method. *P < 0.05, **P < 0.01, ***P < 0.001.

The specific antibody subclasses induced by different SPn vaccines varied markedly, and the dominant Ig subclass correlating with OPA also differed. To investigate the functional relevance of the antibody responses induced by PBPV, we first assessed the correlation between the 4 specific antibodies and the OPA indices of 6 serotypes using multiple correlation coefficient analysis. Notably, serotype 4 exhibited a strong and sustained association at D30 (*R* = 0.675), D90 (*R* = 0.737), and D180 (*R* = 0.748) (Figure 5A). In contrast, only serotype 11A showed a weak correlation (*R* = 0.4) with Ply-specific antibody titers (Supplemental Figure 7A). Combined with the earlier ELISA results (Figure 1, A and B), these findings suggest that the Ply-specific antibodies induced by PBPV are insufficient to effectively correlate with OPA.

Based on these observations, we further evaluated the influence of PspA-specific antibody subclasses on OPA by performing Spearman’s correlation analysis between the OPA indices of different serotypes and the titers of P3296-, P5668-, and PRx1-specific antibody subclasses. Notably, the IgG titers of P3296, P5668, and PRx1 showed a significant correlation with the OPA indices of serotype 38F. Similarly, P3296 and PRx1 IgG titers exhibited an association with the OPA indices of serotype 6A, while P3296 IgG titers alone correlated significantly with the OPA indices of serotypes 4 and 11A. Interestingly, the OPA indices of serotype 14 was more strongly associated with IgM titers of the 3 PspA proteins rather than IgG titers, indicating that IgG-correlated OPA is markedly more potent than IgM-correlated OPA. Additionally, the dominant IgG subclass correlating with OPA varied across serotypes and proteins: (a) for P3296, the OPA indices of serotypes 6A, 38F, and 4 were significantly correlated with IgG1 titers (*R* = 0.509, 0.426 and 0.454). (b) For P5668, the IgG3 titers were correlated with the OPA indices of serotypes 6A and 11A (*R* = 0.575 and 0.481). (c) For PRx1, the OPA indices of serotypes 6A, 38F, and 4 were significantly correlated with the IgG2 titers (*R* = 0.575, 0.612, and 0.496) (Figure 5, B–D, and Supplemental Figure 7B).

Furthermore, the degree and number of correlations between the OPA indices of the 6 SPn serotypes and the titers of the 3 protein-specific antibodies (P3296, P5668, and PRx1) gradually decreased over time (Figure 5E and Supplemental Figure 8, A and B). Surprisingly, the P3296-specific IgG and IgG1 titers remained strongly associated with the OPA index of serotype 4 even at D180 (Figure 5B and Supplemental Figure 8, A and B). These results highlight the complex and dynamic nature of antibody-correlated OPA, with distinct roles for different antibody subclasses and proteins in correlating functional immune protection.

### The dominant antibody subclasses correlating with ADNKA, ADCP, and ADNP varied across different PspA-specific antibodies.

Antibodies bind epitopes via Fab regions and engage Fc receptors on immune cells through their Fc regions, correlating functions such as antibody-dependent NK cell activation (ADNKA), antibody-dependent cellular phagocytosis (ADCP), and antibody-dependent neutrophil phagocytosis (ADNP). While PspA-specific antibodies induce OPA, their roles in ADNKA, ADCP, and ADNP remain less explored. Investigating the contributions of P3296-, P5668-, and PRx1-specific antibodies and their subclasses to these functions not only reveals their broader immune roles but also provides a more comprehensive understanding of the PBPV-induced immune response in humans.

**Figure 6:**
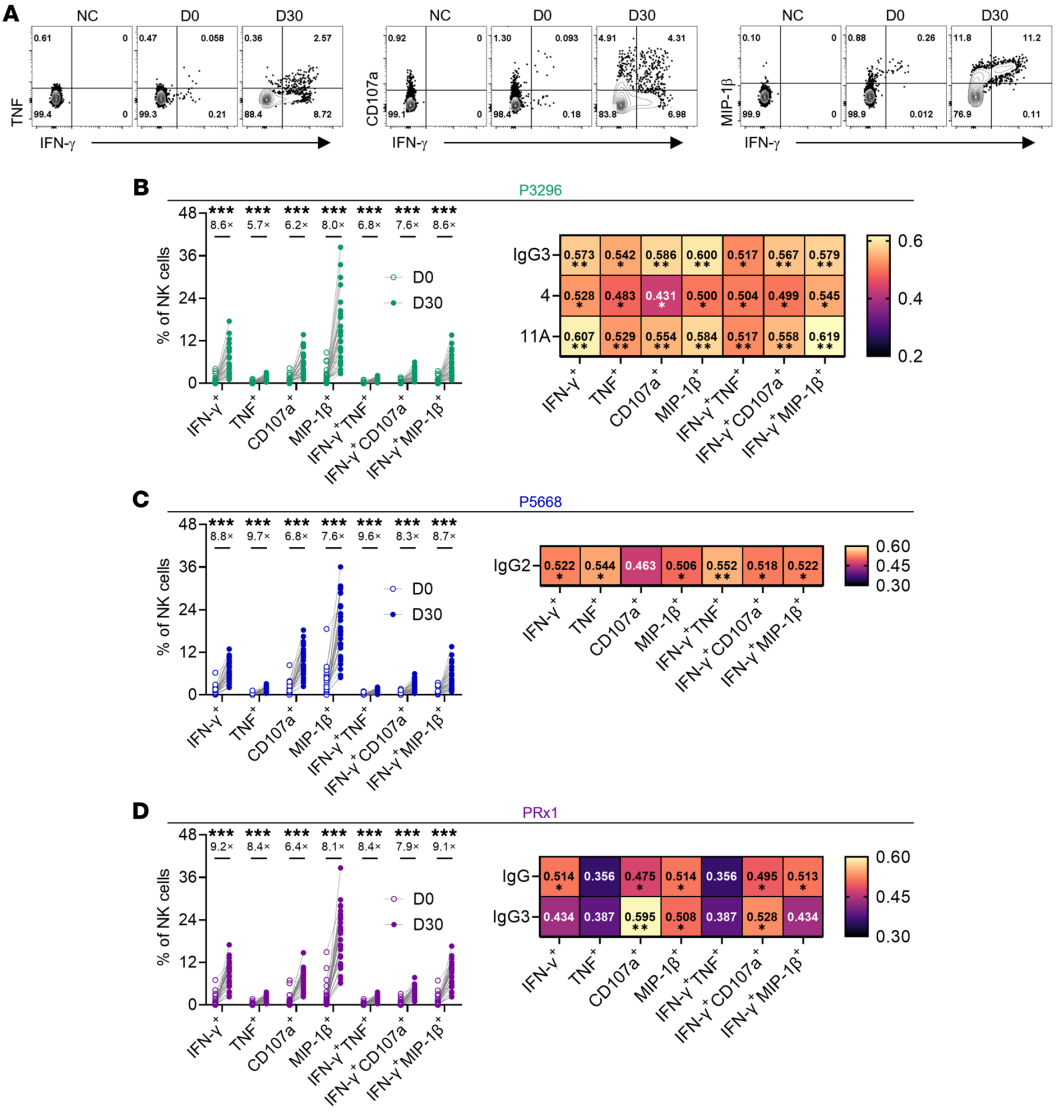
Analysis of dominant subclasses of different PspA-specific antibodies in inducing NK cell activation. (**A**) Representative flow cytometry dot plots. Responses include the secretion of IFN-γ, TNF, and MIP-1β and the expression of CD107a. NC, Negative Control, i.e., replacing the sample serum with Dulbecco’s PBS; all other operational procedures are consistent with those of the experimental group. (**B**–**D**) The left part presents the corresponding statistical analyses of NK cell responses to the 3 PspA proteins. The right part presents the correlation analysis linking NK cell effector functions with antibody titer and the OPA indices for the 6 serotypes. Significance was measured using Wilcoxon’s rank-sum test and Spearman’s correlation analysis in **B**–**D**. All *P* values were adjusted for multiple testing using the BH method. *P < 0.05, **P < 0.01, ***P < 0.001.

To assess the ADNKA-inducing capacity of P3296-, P5668-, and PRx1-specific antibodies, we performed an in vitro NK cell–antibody coculture assay. Compared with baseline (D0), D30 antibodies markedly enhanced NK cell secretion of IFN-γ, TNF, CD107a, and MIP-1β, with similar responses across the 3 protein-specific antibody groups (Figure 6, A–D). Correlation analysis revealed distinct dominant antibody subclasses correlating with ADNKA: P3296- and PRx1-specific IgG3 showed strong correlations, while P5668-specific IgG2 showed the strongest association (Figure 6, B–D, and Supplemental Figure 9, A and B). Notably, P3296-specific ADNKA correlated with OPA indices of serotypes 4 and 11A (Figure 6B and Supplemental Figure 9, A and B). These findings highlight the differential mechanisms of NK cell activation by PspA-specific antibodies and their potential contribution to OPA of specific serotype.

**Figure 7:**
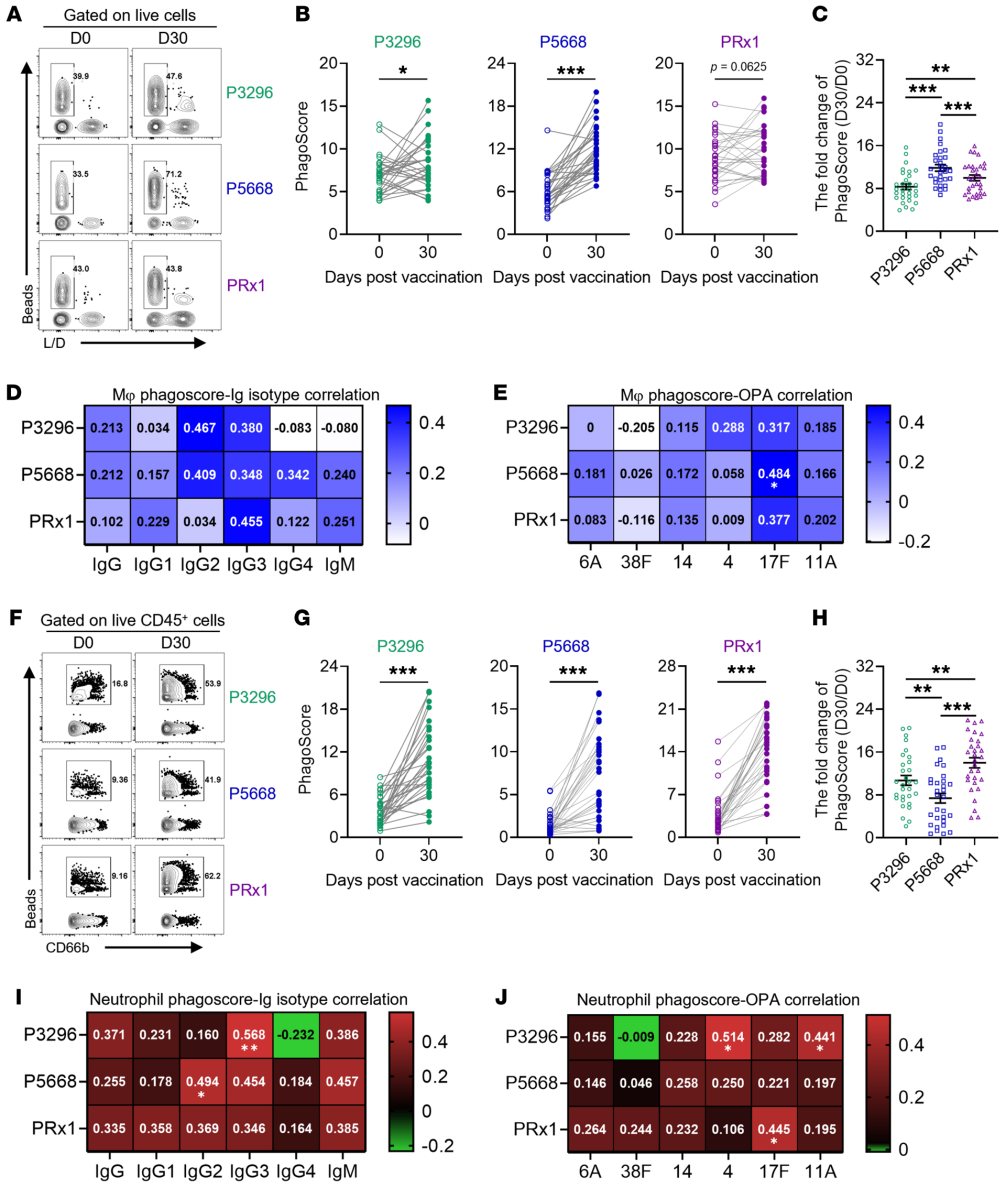
ADCP and ADNP induced by PBPV. (**A** and **F**) Representative flow cytometry dot plots illustrating the phagocytic ability of THP-1/neutrophil induced by PBPV. (**B** and **G**) The phagocytic capacity of THP-1/neutrophils induced by P3296, P5668, and PRx1 at D0 and D30. (**C** and **H**) Fold change in phagocytic capacity of THP-1/neutrophils induced by P3296, P5668, and PRx1 at D30. Data are presented as mean ± SEM. (**D** and **I**) Correlation analysis between phagocytic ability of THP-1/neutrophil and antibody titers against P3296, P5668, and PRx1. (**E** and **J**) Correlation analysis between phagocytic ability of THP-1/neutrophil and the OPA indices of 6 serotypes (6A, 38F, 14, 4, 17F, and 11A). Significance was measured using Wilcoxon’s rank-sum test in **B**, **C**, **G**, and **H** and Spearman’s correlation analysis in **D**, **E**, **I**, and **J**. All *P* values were adjusted for multiple testing using the BH method. *P < 0.05, **P < 0.01, ***P < 0.001.

We further evaluated the ability of P3296-, P5668-, and PRx1-specific antibodies to correlate with ADCP. While P3296- and P5668-specific antibodies markedly enhanced THP-1 cell phagocytosis of their respective target proteins, PRx1-specific antibodies lacked this functionality (Figure 7, A and B). Among the 3, P5668-specific antibodies demonstrated the strongest ADCP-inducing capacity, followed by PRx1, with P3296 showing the weakest activity (Figure 7C). However, when we attempted to identify the potential IgG antibody subclasses that possibly correlate with ADCP through correlation analysis, we did not detect any significant correlations (Figure 7D). In addition, only P5668-specific ADCP correlated with OPA index against serotype 17F (*R* = 0.484) (Figure 7E), suggesting that P5668-specific antibodies may preferentially correlate phagocytic killing of this serotype through monocytes rather than neutrophils.

In assessing ADNP induction, P3296-, P5668-, and PRx1-specific antibodies all markedly enhanced neutrophil phagocytosis (Figure 7, F and G), with PRx1-specific antibodies demonstrating the strongest activity (Figure 7H). Correlation analysis revealed distinct dominant antibody subclasses correlating with ADNP: P3296-associated ADNP correlated with IgG3 (*R* = 0.568) and P5668 with IgG2 (*R* = 0.494; Figure 7I), indicating variability in subclass dominance across proteins. Furthermore, ADNP induced by P3296 correlated with OPA against serotypes 4 and 11A (*R* = 0.514 and 0.441), while PRx1-associated ADNP only correlated with serotype 17F (*R* = 0.445) (Figure 7J). Combined with earlier OPA results, these findings suggest that neutrophil phagocytosis correlated by PspA-specific antibodies may be partially diminished without complement involvement, highlighting the critical role of complement activation in enhancing neutrophil-correlated immune responses.

## Discussion

Our research has been focused on developing a SPn vaccine by utilizing combinations of multiple pneumococcal proteins as an alternative to traditional capsular polysaccharides. Beyond the traditional approach of monitoring antibody titer kinetics, we utilized a comprehensive evaluation framework employing a broad array of functional antibody assays to assess the efficacy of the PBPV over 0–180 days after vaccination, which provided robust evidence of PBPV’s vaccine-elicited immunity over time. These included not only the widely used OPA assay but also Ply neutralization, ADNKA, ADCP, and ADNP. This multifaceted approach allowed us to capture the full spectrum of immune responses elicited by PBPV. The kinetic analysis revealed that antibodies against P3296, P5668, and PRx1 peaked at D30 and remained elevated throughout the study period, demonstrating sustained immunogenicity. In contrast, Ply-specific antibodies showed weaker induction and limited persistence, suggesting potential areas for optimization. However, a phase Ia trial revealed that repeated administration of Ply in individuals aged 18–49 years enhanced both Ply antibody titers and neutralizing efficacy (24). This immunogenicity profile suggests the potential for multiple PBPVs to similarly improve Ply-neutralizing capacity in 50- to 69-year-olds, warranting further clinical investigation. By integrating these diverse functional assays, we provide a more holistic understanding of PBPV’s immune efficacy, highlighting its potential as a next-generation pneumococcal vaccine.

Compared with the traditional polysaccharide vaccine PPV23, PBPV offers several marked advantages. First, PBPV provides broader cross-serotype protection by targeting conserved pneumococcal proteins rather than serotype-specific capsular polysaccharides. The inclusion of key target proteins such as P3296, P5668, PRx1, and Ply in the PBPV formulation ensures coverage across nearly all pneumococcal serotypes, addressing the limitations of incomplete coverage and serotype replacement associated with PPV23. Our results showed that PBPV-induced OPA against serotypes 6A and 38F was markedly stronger than that of PPV23, and this advantage was maintained up to D90, demonstrating its superior and durable immune efficacy. Second, PBPV’s efficacy was not influenced by age or gender, making it a versatile option for diverse populations, including the elderly and individuals who are immunocompromised. This is particularly important given the reduced immune responsiveness observed in older adults, who are at higher risk of pneumococcal infections. Third, the production process of PBPV is simpler and more cost-effective than that of PPV23. While PPV23 requires complex extraction and purification of capsular polysaccharides from multiple serotypes, PBPV is based on recombinant protein technology, which is more scalable and lowers the industrial production threshold (26). This simplification not only reduces manufacturing costs but also accelerates vaccine development and deployment, making PBPV a more accessible option for global immunization programs. Additionally, PBPV’s protein-based design allows for easier modification and optimization, enabling rapid adaptation to emerging pneumococcal strains. Moreover, sequence modifications can be designed to minimize similarity with human self-antigens, thereby reducing the risk of immune-mediated damage or autoimmunity. This adaptability makes PBPV a highly versatile platform for vaccine development. In contrast, expanding the serotype coverage of PPV23 would require the addition of more capsular polysaccharides, which not only increases production complexity but may also lead to reduced immunogenicity for individual serotypes due to antigen competition (7, 27). In summary, PBPV surpasses PPV23 with broader protection, simpler production, and greater adaptability, making it a superior choice for global immunization.

Our systematic evaluation reveals that the functional architecture of PBPV-induced immunity is fundamentally shaped by 2 interdependent factors: antigen-correlated IgG subclass polarization and adjuvant-correlated modulation of effector responses. The distinct protective mechanisms associated with each vaccine component — P3296-primed IgG1 for OPA and IgG3 for ADNKA and ADNP; P5668-induced IgG2 for ADNKA and ADNP, as well as IgG3 for OPA; and PRx1-enhanced IgG2 for OPA and IgG3 bridging ADNKA — collectively establish a multilayered defense network. Notably, this subclass specialization correlated with serotype-specific vulnerability profiles: encapsulated serotypes relying on IgG1-correlated OPA versus nonencapsulated variants requiring IgG2-correlated ADCP. Such functional stratification aligns with emerging paradigms in antibacterial immunity, where Fc-mediated effector mechanisms compensate for complement deficiencies in specific host populations.

Our study elucidates the association between antibody subclasses and distinct antibacterial immune responses, which reveals a dynamic antibody landscape. This finding thereby lays the foundation for optimizing vaccine components through strategic modulation of adjuvant selection, antigenic protein ratios, and other parameters, crucially highlighting that adjuvant deployment substantially dictates both the immunoprotective efficacy and the antibody repertoire composition of the final vaccine formulation (28). For example, aluminum hydroxide formulations skewed responses toward IgG1/IgG3 (Th2 bias), optimal for pediatric populations requiring robust complement activation (29, 30). In contrast, TLR-agonist adjuvants (e.g., CpG) enhanced IgG2 production while preserving ADCP activity in elderly subjects (31). These observations underscore the need for age-stratified adjuvant strategies, particularly given PBPV’s demographic-neutral seroprotection rates. While our findings position PBPV as a transformative pneumococcal vaccine candidate, 2 limitations warrant attention: (a) the current formulation shows reduced IgG4 induction (<1%), potentially impacting mucosal immunity longevity; and (b) antigenic interference between Ply and PspA components may require dose optimization. Future studies should explore chimeric PspA-Ply fusion proteins to enhance epitope synergy while minimizing interference. Therefore, to fully realize PBPV’s potential, it is necessary to align antigen composition with regional serotype prevalence and tailor adjuvants to host immunocompetence, which enables precision vaccination strategies unattainable with conventional approaches.

Pneumococcal protein vaccines target key virulence and adhesion factors, offering potential for broad, serotype-independent protection. Besides multiprotein combination vaccines like PBPV, numerous pneumococcal proteins have been explored as immunogens in experimental vaccine studies (13). Pneumococcal Surface Protein C and Choline-Binding Protein A are promising due to their roles in bacterial colonization and virulence, but their high variability complicates vaccine design (32, 33). Pneumococcal Neuraminidase A and Endopeptidase O are essential virulence factors, yet their weak immunogenicity often necessitates adjuvants to enhance efficacy (34, 35). Moreover, these pneumococcal protein vaccines still lack sufficient clinical data and evaluation protocols to reflect their true vaccine efficacy. In contrast, the PBPV leverages multiple conserved proteins, reducing reliance on polysaccharide antigens and addressing the limitations of variability and immunogenicity seen in single-protein vaccines. By targeting diverse virulence mechanisms, PBPV offers a comprehensive approach to pneumococcal disease prevention, making it a promising alternative to traditional vaccines. Future advancements in antigen selection and adjuvant technology will further enhance its potential.

There are several limitations in our study. First, the cohort size was relatively small. Second, the long-term durability of PBPV-induced immune responses remains to be evaluated. Third, while we explored the functional roles of IgG subclasses, the mechanisms underlying their differential effects on OPA, ADNKA, ADNP, and ADCP require further investigation. Fourth, our study lacks efficacy data, which is essential for validating the protective effect of PBPV. Future studies should address these limitations by expanding the cohort, extending the follow-up period, and exploring the molecular basis of antibody-correlated immunity. Overall, our study demonstrates that PBPV is a highly promising candidate for replacing traditional polysaccharide vaccines like PPV23. Its ability to elicit broad-spectrum protection, overcome age- and gender-related limitations, and simplify vaccine production makes it a viable alternative for global use. Furthermore, the flexibility to optimize target protein composition and adjuvant selection provides opportunities to enhance its immune efficacy. With further refinement, PBPV has the potential to become a cornerstone of pneumococcal vaccination strategies, offering improved protection against this substantial public health threat.

## Methods

### Sex as a biological variable.

The study included both male and female participants, and similar findings are reported for both sexes.

### Cohort and sample.

Serum samples were collected from 60 volunteers (25 female and 35 male) at D0, D30, D90, and D180 in a phase Ib, randomized, double-blind clinical trial conducted collaboratively by the Guangzhou Laboratory and CanSino Biologics Inc. 30 volunteers received a single dose of PBPV, while the remaining 30 volunteers received a single dose of PPV23 as the control group. This study is registered at ClinicalTrials.gov (NCT05622942).

### Vaccine.

Each 0.5 mL dose of PBPV contained 40 μg P3296, 40 μg P5668, 48 μg PRx1, and 64 μg Ply. Each 0.5 mL dose of PPV23 contained 25 μg of capsular polysaccharides from each of the following serotypes: 1, 2, 3, 4, 5, 6B, 7F, 8, 9N, 9V, 10A, 11A, 12F, 14, 15B, 17F, 18C, 19A, 19F, 20, 22F, 23F, and 33F.

### Measurement of antigen-specific antibody titers by subclass.

ELISA was used to measure antigen-specific antibody responses for multiple subclasses. 50 ng of P3296, 50 ng of P5668, 400 ng of PRx1, and 400 ng of Ply were coated onto an ELISA plate as antigens and incubated for 16 hours. The plate was then blocked with 200 μL of blocking solution containing 5% BSA for 2 hours. Serum samples were diluted in a 2-fold gradient, and after blocking, 100 μL of the serum dilutions was added to the ELISA plate. PBS containing 1% BSA was used as a negative control. The plate was incubated for 1.5 hours, followed by the addition of HRP-conjugated antihuman IgG, IgG1, IgG2, IgG3, IgG4, and IgM secondary antibody and incubation for 1 hour. The reaction was developed using TMB substrate for 10 minutes, and the reaction was stopped by adding stop solution. OD was measured at 450 nm using a Biotek ELISA reader. Data analysis was performed using a 4-parameter nonlinear regression model in GraphPad Prism 9 software.

### Ply neutralization assay.

A Ply-neutralizing test assay was used to assess the neutralizing activity of Ply at D0, D30, D90, and D180. Prior to the assay, serum samples were inactivated by incubation at 56°C for 30 minutes. The serum samples were inactivated during processing and subsequently diluted with saline as the diluent for the initial n-fold dilution, followed by 2-fold gradient serial dilutions. Hemolysis inhibition of 100% (100 μL saline), 0% (100 μL purified water), and Ply hemolysis control (50 μL saline and 50 μL Ply) were used as controls. For the Ply neutralization assay, Ply was diluted to a defined concentration, and the diluted serum was added to all wells except the controls. Subsequently, 100 μL of 2% rabbit erythrocytes was added to all wells, followed by tapping the plate to mix the liquid. The plate was then incubated at 37°C for 60 minutes. After incubation, the plate was centrifuged at 1,300*g* for 10 minutes. A 100 μL aliquot of the supernatant was transferred to the corresponding wells of a new 96-well microtiter plate, and the OD at 450 nm was measured.

### Measurement of OPA index.

The OPA assay was performed according to a previously published standard procedure (36). Prior to the assay, serum samples were inactivated by incubation at 56°C for 30 minutes. Serum were prepared in a 96-well plate with a triple-gradient dilution (serum type 6A was preadsorbed with sugar), followed by the addition of 10 μL of bacterial suspension (serotypes 6A, 38F, 4, 14, 17F, and 11A) for incubation under shock for 30 minutes. HL60 cells (purchased from ATCC, clone CCL-240) were differentiated into granulocytes by culturing in RPMI-1640 medium supplemented with 10% FBS, 1% l-glutamine, and 0.8% dimethylformamide at an initial density of 4 × 10^5^ cells/mL for 5–6 days (37). The differentiated HL60 cells were combined with rabbit complement at a 4:1 ratio and added to 96-well plates at 50 μL per well. The plates were incubated at 37°C with 5% CO_2_ for 75 minutes. After incubation, 10 μL of the mixture was removed from each well and inoculated onto a THYA plate (Todd-Hewitt broth supplemented with 0.5% yeast extract and 1.5% agar). After thoroughly drying, a layer of overlay agar containing the appropriate antibiotics and triphenyl tetrazolium chloride was poured onto the plate. Once completely solidified, the plates were incubated at 37°C with 5% CO_2_ for 16–18 hours. Finally, a colony counter was used to count the number of bacteria corresponding to each serum dilution. The OPA index was analyzed using Opsotiter 3 software. The OPA index was defined as the serum dilution that killed 50% of the bacteria.

### OPA titer ranking methods.

The 3 antibody response quantification strategies developed by Ravichandran et al. (25) were used: rank, strength, and extent. Individuals in a cohort were ranked based on vaccine responsiveness across all serotypes using the dense ranking method (25). For each serotype, participants were assigned a dense rank according to their fold change in OPA index levels. Participants with the same fold change levels received the same rank, with the next participants getting the subsequent rank. These individual serotype ranks were then summed to obtain an overall score for each individual. Dense ranking was then applied to these overall scores to determine the final rank for each individual. Individuals with higher fold changes in titer levels across many serotypes ranked higher than others, while those with lower levels across many serotypes received lower ranks. This ranking strategy is robust to outliers (that is, a participant with very high titer levels only for 1 serotype) and is based on a multivariate approach (using 6 serotypes) to quantify vaccine responsiveness of individuals. Strength represents the sum of fold change in OPA titer levels across all serotypes and explains the dynamics of baseline and postvaccination changes per sample. Higher scores indicate stronger responses in individuals. An OPA titer of 2 or above was considered a significant response to a specific serotype, forming the basis for the extent strategy. Extent measures the number of serotypes (out of 6) an individual responds to significantly, with higher values indicating a broader response. These measures have unique advantages. To compute the baseline-adjusted fold change for the 6 serotypes, the maxRBA function (titer package in R) with scoreFun set to “sum” was used. The adjusted fold change was then used to rank individuals through a dense ranking approach. The adjusted fold change was then used to rank individuals through a dense ranking approach.

### Antibody-dependent NK cell activation.

Antibody-dependent NK cell activation and degranulation were measured as previously described (38). ELISA plates (Corning) were coated overnight at 4°C with PspA-3296, PspA-5668, and PspA-PRx1 (0.5 mg/mL) and then blocked with 5% BSA in PBS for 2 hours at 37°C. 100 μL of serum diluted 1:10 or Dulbecco’s PBS (as a negative control) was added to each well and incubated for 2 hours at 37°C. NK cells were isolated from PBMCs of healthy donors using the NK Cell Enrichment Kit (STEMCELL Technologies) and rested in IL-2 (20 U/mL) for 5 hours at 37°C. Isolated NK cells were premixed with a cocktail containing anti–CD107a-BV785 (BioLegend, clone LAMP-1, catalog 121641) and brefeldin A (1 μM, BD Biosciences) and added to the wells. The mixture was incubated at 37°C for 5 hours. Following incubation, cells were stained for surface markers using anti–CD56-BB515 (BD Pharmingen, clone B159, catalog 564488) and anti–CD16-APC (BD Pharmingen, clone 3G8, catalog 561248). Intracellular markers were stained with anti–macrophage inflammatory protein-1β (MIP-1β)-V450 (BD Pharmingen, clone D21-1351, catalog 561282), anti–IFN-γ-PE (BD Pharmingen, clone B27, catalog 554701), and anti–TNF-BUV737 (Invitrogen, clone MAb11, catalog367-7349-42). NK cells were gated outside the CD16^–^CD56^–^ cell population. The expression levels of CD107a, IFN-γ, TNF, and MIP-1β were detected by flow cytometry.

### Antibody-dependent cellular phagocytosis.

Monocyte phagocytosis was evaluated as previously described (39). PspA-3296, PspA-5668, and PspA-PRx1 were biotinylated using EZ-Link NHS long-chain biotin (Thermo Fisher Scientific) according to the manufacturer’s instructions. The biotinylated proteins were then adsorbed onto 1 μm FluoSpheres NeutrAvidin beads (Invitrogen) at a 1:1 ratio of biotinylated protein/beads. 10 μL of a 1:100 suspension of antigen-coupled beads was added to each well of a 96-well plate, along with an equal volume of serum diluted 1:10. The plates were incubated at 37°C for 2 hours and washed with PBS. 25,000 THP-1 cells (ATCC TIB-202 human acute monocytic leukemia cell line) were added and incubated at 37°C for 18–20 hours. Cells were incubated with Live/Dead FVS440 for 15 minutes and stained for surface markers using anti–CD14-APC-Cy7 (BD Pharmingen, clone MfP9, catalog 560180). They were then fixed and permeabilized using the FIX & PERM Cell Permeabilization Kit (Thermo Fisher Scientific), and phagocytosis was measured by a FACSFortessa (BD Biosiences). Phagocytosis scores were calculated as (%beads − positive cells) × (geometric MFI)/10,000.

### Antibody-dependent neutrophil phagocytosis.

Neutrophil phagocytosis was evaluated as previously described (40). Briefly, as described for cellular phagocytosis, PspA-3296, PspA-5668, and PspA-PRx1 were biotinylated and coupled to 1 μm fluorescent neutravidin beads. 10 μL of a 1:100 dilution of coupled beads was opsonized with 10 μL of serum diluted 1:25 at 37°C for 2 hours. Neutrophils were isolated from healthy donors’ whole blood using the RosetteSep Neutrophil Enrichment Kit (STEMCELL Technologies). 50,000 isolated leukocytes were added per well and incubated for 1 hour at 37°C. The cells were then stained with APC antihuman CD66b antibody (BioLegend, clone G10F5, catalog 305118), fixed, and permeabilized using the FIX & PERM Cell Permeabilization Kit. Phagocytosis was measured by a FACSFortessa. Phagocytosis scores were calculated as (%beads − positive cells) × (geometric MFI)/10,000. Cells were gated on live CD66b^+^ populations, and phagocytosis scores were calculated as described previously.

### Flow cytometry.

Flow cytometry data were collected on a BD LSRFortessa X-20 instrument (BD Biosciences). Standardized SPHERO rainbow beads (Spherotech) were used to track and adjust photomultiplier tubes over time. UltraComp eBeads (Thermo Fisher Scientific) were used for compensation. Data were analyzed using FlowJo v10 (BD Biosciences).

### Correlation analysis.

Antibody titers of subclasses, the OPA indexes at D30, D90, and D180 time points in the PBPV-vaccinated population, and the ADNKA, ADCP, and ADNP response data at D30 were collected. SPSS software was used to perform Spearman’s correlation analysis between the following pairs: antibody titers with the OPA indices at D30, D90, and D180; antibody titers with ADNKA; antibody titers with ADCP; antibody titers with ADNP; OPA indices with ADNKA; OPA indices with ADCP; and OPA indices with ADNP. The resulting *R* values were visualized in a heatmap, with statistical significance indicated by asterisks. All *P* values were adjusted for multiple testing using the Benjamini–Hochberg (BH) method.

### Statistics.

Data analysis was performed using GraphPad Prism and R. Statistical significance was set at *P* < 0.05 (*P* < 0.05, *P* < 0.01, *P* < 0.001). Comparisons among the D0–D180 time points were performed using Wilcoxon’s signed-rank test or paired *t* test (2 tailed), allowing pairing of samples from the same individuals. Comparisons between the PBPV and PPV23 groups were performed using Mann-Whitney test, allowing unpairing of samples from different individuals. All tests were followed by BH correction for multiple comparisons. Antibody titers, OPA indices, and fold changes are presented as geometric means titer with the 95% CI, and positivity rates are presented as mean ± SEM. Linear regression analysis was conducted with GraphPad Prism. *P* values for whisker-and-box plots were calculated using 2-sided Wilcoxon’s rank-sum tests using the stat_compare_means function from the ggpubr package (v0.4.0).

### Study approval.

This study was approved and supervised by The First Affiliated Hospital of Guangzhou Medical University Ethics Committee (2021–78). All participants provided written informed consent before participating in this study.

### Data availability.

All data supporting the findings of this study are included in this article and the Supporting Data Values file and are available from the corresponding author upon reasonable request.

## Author contributions

ZW, JW, and KL designed the experiments and analyzed the data. KL, JY, X Zhai, YW, XS, XY, SJ, X Zhou, and YZ performed the experiments and analyzed the data. JG, XS, and BW recruited the cohort and carried out clinical treatments. ZW, JW, and TZ reviewed this work. ZW and JW wrote the manuscript. All authors have read and approved the manuscript. The order of the co–first authors KL, JY, and X Zhai was determined by their contributions to this project.

## Funding support

- National Key Research and Development Program of China (2022YFC2604104 and 2023YFC2306400).
- National Natural Science Foundation of China (82271801 and 82341244).
- 2025 Special Project of Basic and Applied Basic Research (Young Doctors ‘Qihang’ Project) (2025A04J3556).
- Major Project of Guangzhou National Laboratory (GZNL2023A01009).
- Research and Development Program of Guangzhou National Laboratory (SRPG23-005).
- Science and Technology Program of Guangzhou Laboratory (SRPG22-006).

## Supplementary Material

Supplemental data

Supporting data values

## Figures and Tables

**Table 2:**
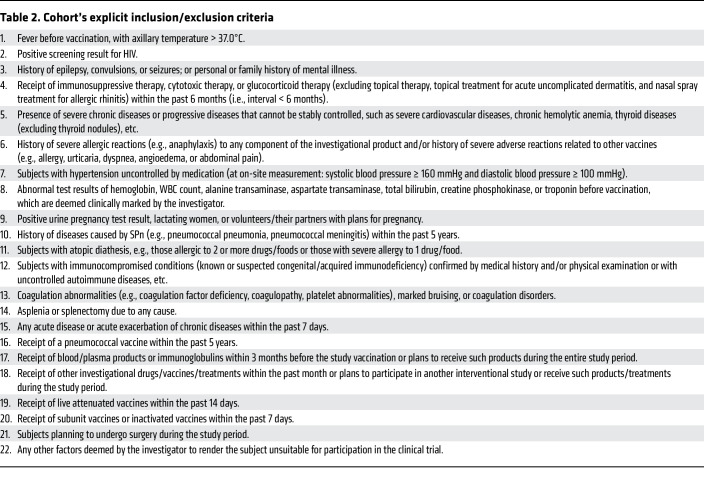
Cohort’s explicit inclusion/exclusion criteria

**Table 1:**
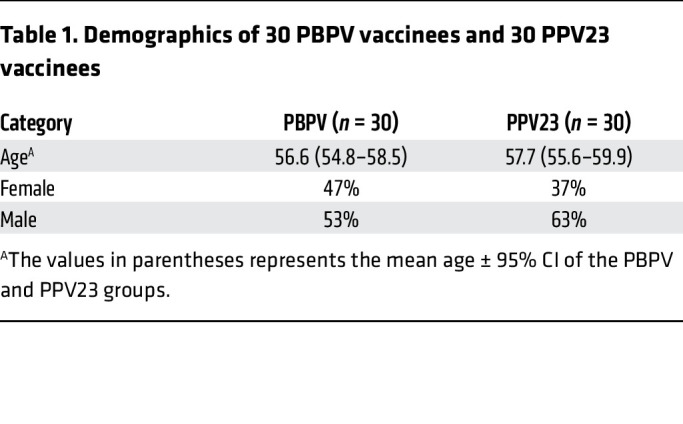
Demographics of 30 PBPV vaccinees and 30 PPV23 vaccinees

## Version 1 02/02/2026

Electronic publication
