# Supplementary material for: A protein-based pneumococcal vaccine elicits broad immunity associated with multifunctional antibody responses in humans
Source: J Clin Invest. 2026 Feb 2;136(3):e196261. doi: 10.1172/JCI196261 (PMC12867161; doi:10.1172/JCI196261)
Supplement: Supplemental data [file jci-136-196261-s067.pdf]

Novel protein-based pneumococcal vaccine elicits broad immunity through IgG subclass dominance and multifunctional antibody responses

Author

Kaiyi Li[1,2], Jinglu Yang[1,2], Xiaobing Zhai[1], Jinbo Gou[3], Xiuwen Sui[3], Bochao Wei[3], Yuan Wang[4], Xiaoling Su[2], Xiaoyun Yang[2], Shiqin Jin[1], Xuan Zhou[1], Yuxuan Zhang[1], Tao Zhu[3], Junxiang Wang[1], Zhongfang Wang[1,2,5]

Affiliations

[1]State Key Laboratory of Respiratory Disease & National Clinical Research Center for Respiratory Disease, Guangzhou Institute of Respiratory Health, the First Affiliated Hospital of Guangzhou Medical University, Guangzhou Medical University, Guangzhou, China.

[2]Guangzhou National Laboratory, Guangzhou, Guangdong, China.

[3] CanSino Biologics Inc., Rongsheng Building, No. 185 South Street, West Area of Dongli Economic and Technological Development Zone, Tianjin, China.

[4]Key Laboratory of Emergency and Trauma of Ministry of Education, Engineering Research Center for Hainan Biological Sample Resources of Major Diseases, The Hainan Branch of National Clinical Research Center for Cancer, the First Clinical College & the First Affiliated Hospital, Hainan Medical University, Haikou 570102.

[5]Shenzhen Hetao Institute of Guangzhou National Laboratory, Guangzhou,

Guangdong, China.
K. L., J.Y. and X.Z. contributed equally to this work
Address correspondence to: Tao Zhu, No. 185 South Street, West District, Tianjin Economic-Technological Development Area, Tianjin, China. Phone:400-922-2099; tao.zhu@cansinotech.com; Junxiang Wang, No. 151 Yanjiang Road, Yuexiu District, Guangzhou, Guangdong Province, China. Phone:15626211053; Email: 120298127@qq.com; Zhongfang Wang, No. 96 Xingdao Huanbei Road, Guangzhou International Bio-Island, Huangpu District, Guangzhou, Guangdong Province, China. Phone: 13929521227; Email: wangzhongfang@gird.cn

Conflict of interest: We declare that we do not have any commercial or associative interests that represent a conflict of interest in connection with the submitted work. Members of CanSino Biologics Inc. in the author list provided substantial support for sample provision, experimental testing, and other related aspects, and they have no financial or other relevant conflicts of interest with this manuscript.

This Supplementary material contains:

Supplemental Figure 1 to 9.

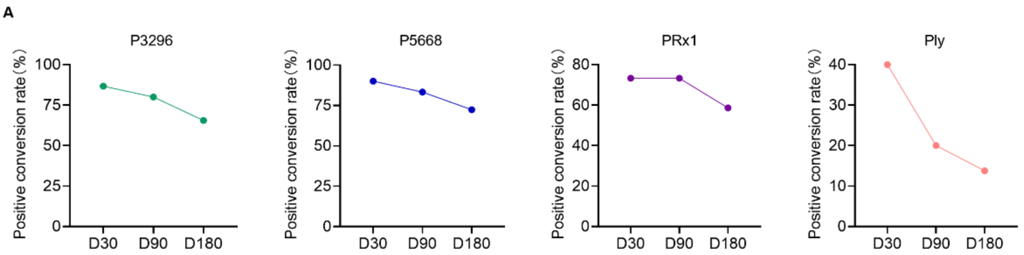

Supplemental Figure 1. The positive conversion rate in the PBPV group. (A) Positive conversion rates for P3296, P5668, PRx1, and Ply were assessed at D30, D90, and D180, positive response defined as a ≥ 4-fold increase in antibody titers compared to D0 levels.

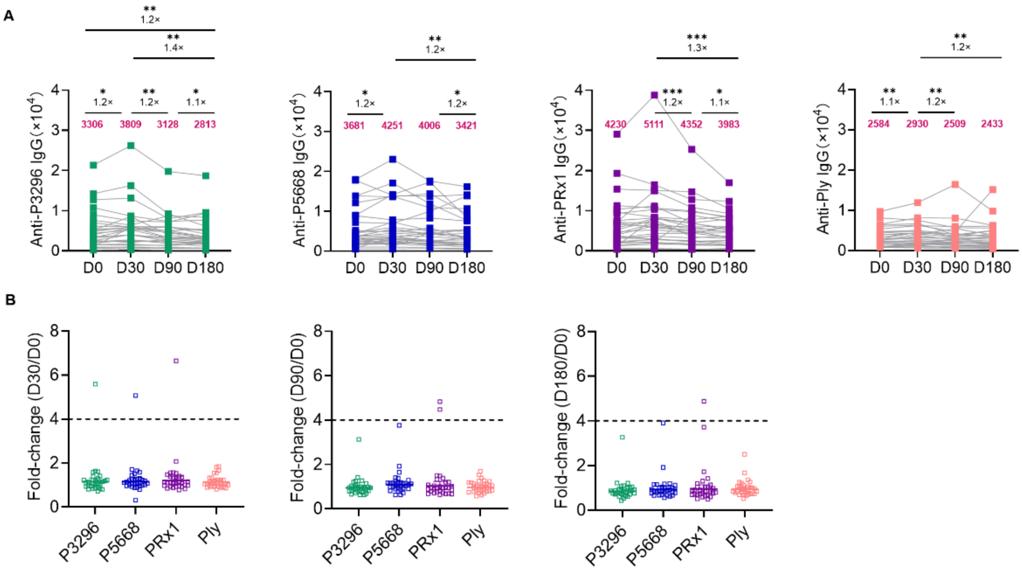

Supplemental Figure 2. PPV23 fails to induce the effective P3296-, P5668-, PRx1- and Ply-specific antibody production. (A) Specific antibody titers against P3296 (green), P5668 (blue), PRx1 (purple), and Ply (pink) were measured in the PPV23 group (n = 30) at D0, D30, D90 and D180. **(B)** Fold changes for P3296, P5668, PRx1, and Ply were assessed at D30, D90 and

D180, defined a ≥ 4-fold increase in antibody titers compared to D0 levels as positive cutoff. The error bars defined geometric mean with 95% CI. The numbers in magenta indicated the geometric mean titers (GMT) in **A**. Significance was measured using the Wilcoxon rank-sum test in **A**. All p-values were adjusted for multiple testing using the Benjamini–Hochberg (BH) method. *p < 0.05, **p < 0.01, ***p < 0.001.

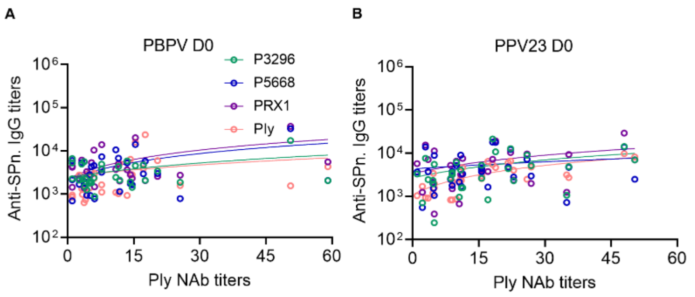

A

	P	R^2
P3296	0.0474	0.1697
P5668	0.0228	0.2278
PRX1	0.0168	0.2579
Ply	0.2832	0.0410

B

	P	R^2
P3296	0.0948	0.1545
P5668	0.3658	0.0293
PRX1	0.0948	0.1539
Ply	0.0004	0.5781

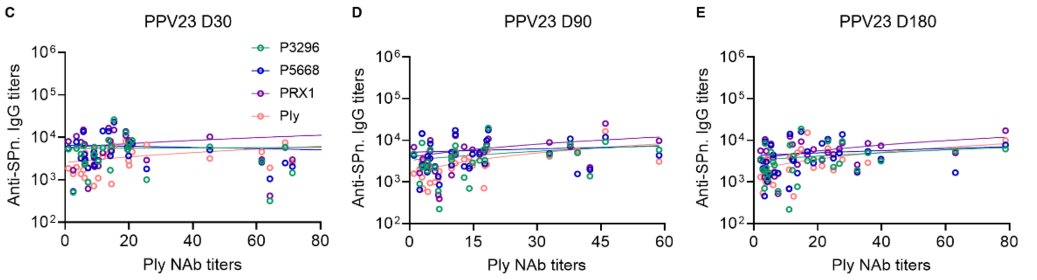

C

	P	R^2
P3296	0.9094	0.0004
P5668	0.8597	0.0077
PRX1	0.3810	0.0604
Ply	0.0440	0.2095

D

	P	R^2
P3296	0.1811	0.0777
P5668	0.5629	0.0121
PRX1	0.0528	0.1641
Ply	0.0056	0.3084

E

	P	R^2
P3296	0.3684	0.0422
P5668	0.5955	0.0208
PRX1	0.0176	0.2550
Ply	0.0176	0.2208

Supplemental Figure 3. Correlation analysis between titers of four protein-specific antibodies and Ply-neutralizing antibody titers induced

by PPV23. (A-E) Correlation analysis between specific antibody titers of the four PBPV immunogens (P3296, P5668, PRx1 and Ply) and neutralizing antibody titers against Ply in the PBPV group at D0 and in the PPV23 group at D0, D30, D90, D180. All p-values were adjusted for multiple testing using the Benjamini–Hochberg (BH) method.

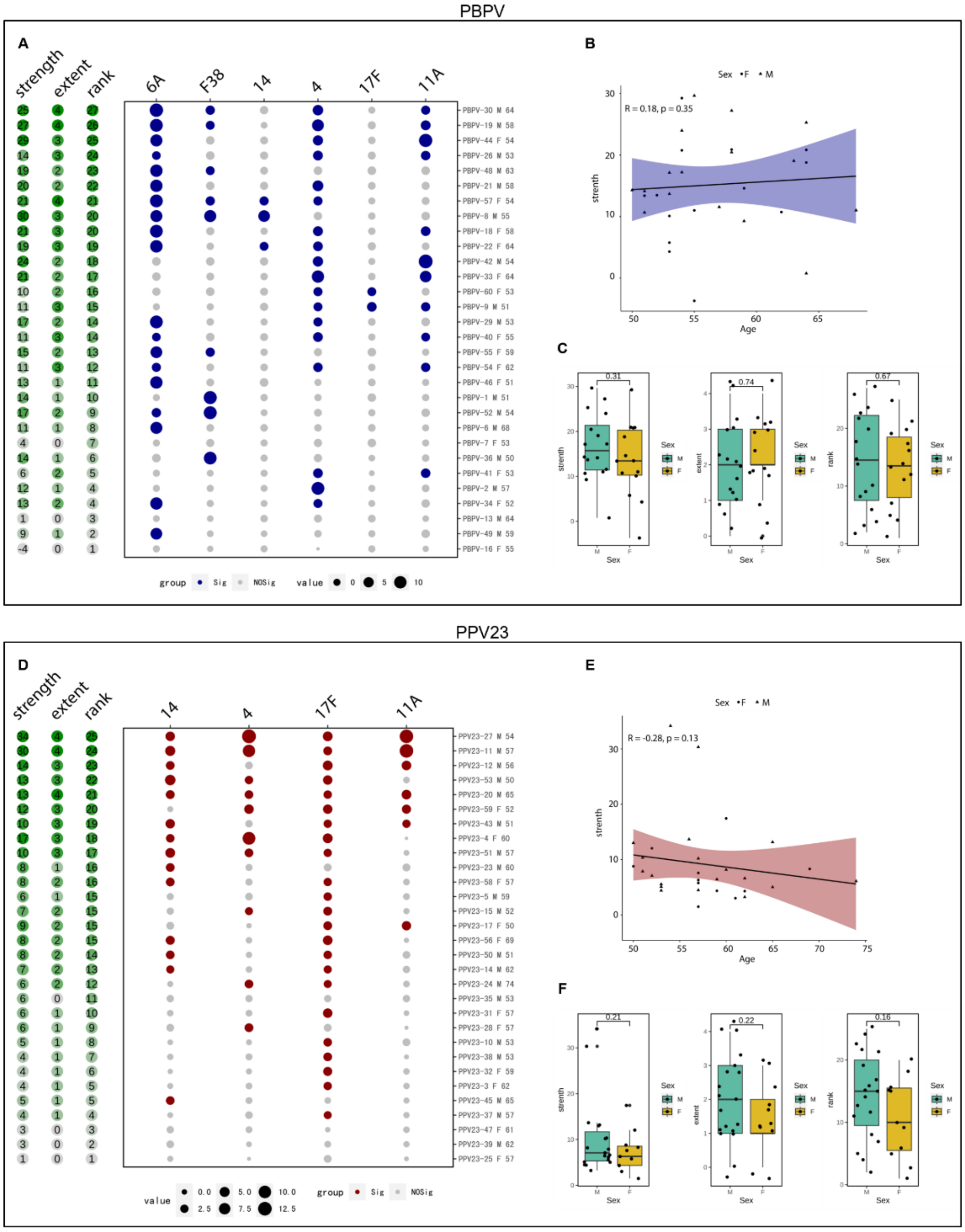

Supplemental Figure 4 Comparative analysis of OPA induced by PBPV and PPV23 at day 90 post-vaccination. (A and **D)** The OPA elicited by PBPV and PPV23 at D90 were presented. The left panel illustrates the strength (log2(sum fold change)), extent, and rank of OPA reactions (strength means the sum of fold change values between baseline and D90 for all serotypes; extent means the number of serotypes (of 4) to which the donor elicits marked (that is, an OPA titer of ≥ 2) responses; rank means an individual's vaccine responsiveness in the cohort based on aggregate responses for all serotypes, where higher ranks represent stronger responses. The middle bubble plot uses color coding to indicate marked responses (Log2 fold change [FC] > 1): blue/red denotes a marked enhancement of OPA functionality (D90/D0 OPA value ≥ 2), while gray indicates a value ≤ 2. Bubble size represents the magnitude of the response. The right panel provides information on participant ID, gender (F for female, M for male), and age. **(B** and **E)** Correlation between OPA response intensity and age at D90 with PBPV and PPV23. **(C** and **F)** Gender-based differences in OPA response intensity, magnitude, and grade at D90 with PBPV and PPV23. Boxplots display the median and interquartile range (IQR, 25–75%), with whiskers indicating values within 1.5 × IQR above and below the quartiles.

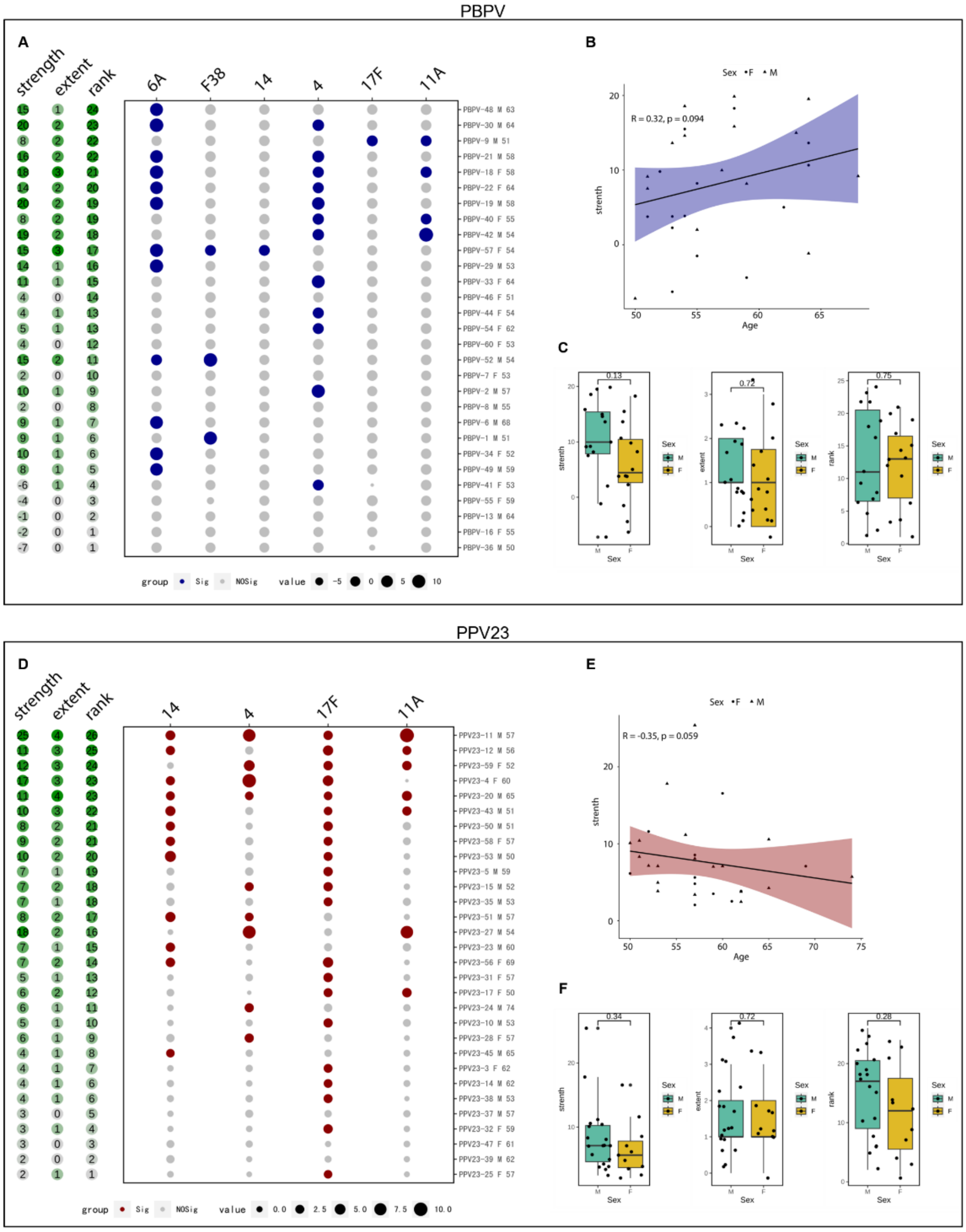

Supplemental Figure 5 Comparative analysis of OPA induced by PBPV and PPV23 at day 180 post-vaccination. (A and **D)** The OPA elicited by PBPV and PPV23 at D180 were presented. The left panel illustrates the strength (log2(sum fold change)), extent, and rank of OPA reactions (strength means the sum of fold change values between baseline and D180 for all serotypes; extent means the number of serotypes (of 4) to which the donor elicits marked (that is,

an OPA titer of ≥ 2) responses; rank means an individual's vaccine responsiveness in the cohort based on aggregate responses for all serotypes, where higher ranks represent stronger responses. The middle bubble plot uses color coding to indicate marked responses (Log2 fold change [FC] > 1): blue/red denotes a marked enhancement of OPA functionality (D180/D0 OPA value ≥ 2), while gray indicates a value ≤ 2. Bubble size represents the magnitude of the response. The right panel provides information on participant ID, gender (F for female, M for male), and age. **(B** and **E)** Correlation between OPA response intensity and age at D180 with PBPV and PPV23. **(C** and **F)** Gender-based differences in OPA response intensity, magnitude, and grade at D180 with PBPV and PPV23. Boxplots display the median and interquartile range (IQR, 25–75%), with whiskers indicating values within 1.5 × IQR above and below the quartiles.

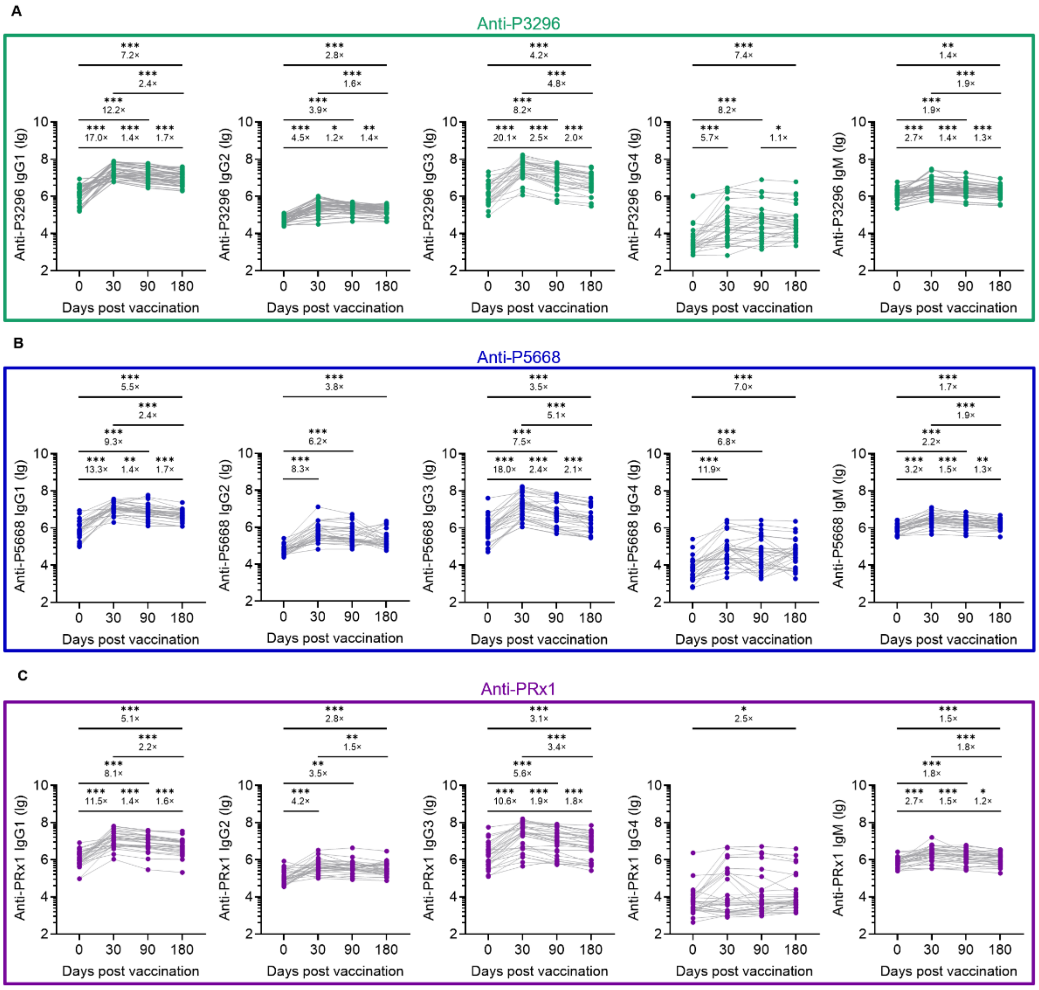

Supplemental Figure 6 Kinetics of P3296-, P5668-, and PRx1-specific antibody subclass titers from day 30 to 180 post-vaccination. (A-C) Titers of antibody subclasses (IgG1, IgG2, IgG3, IgG4, and IgM) induced against P3296, P5668 and PRx1 in vaccinated subjects. Significance was measured using the Wilcoxon rank-sum test in **A-C**. All p-values were adjusted for multiple testing using the Benjamini–Hochberg (BH) method. $^*p < 0.05$, $^{**}p < 0.01$, $^{***}p < 0.001$.

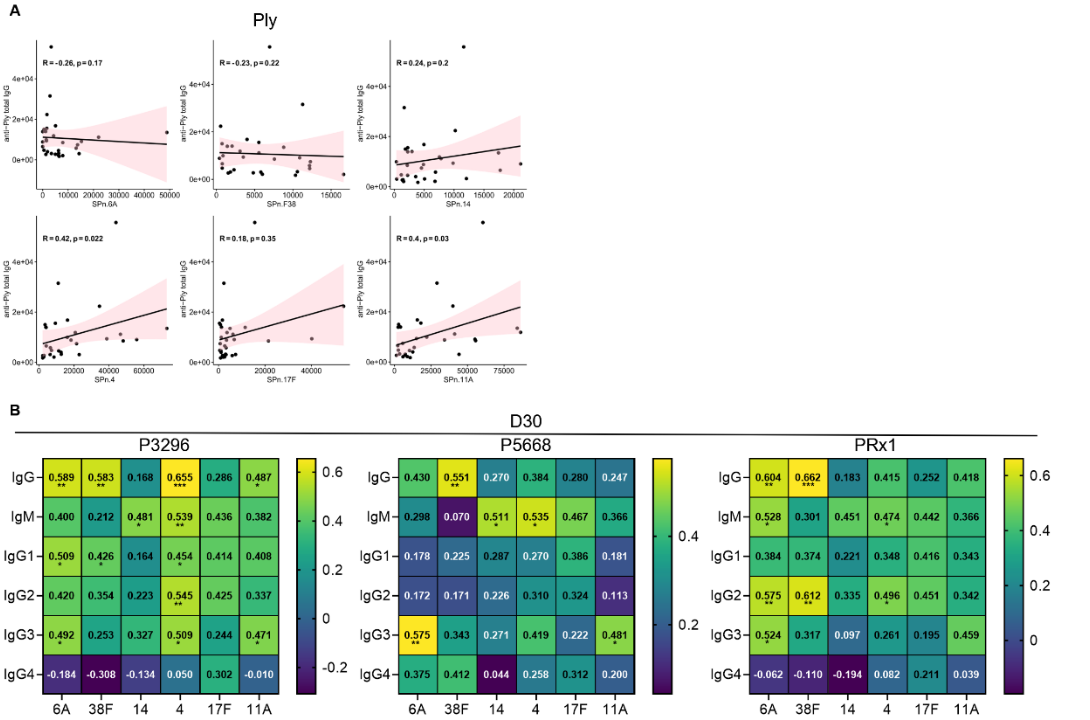

Supplemental Figure 7 Correlation between antibody titers and OPA indices induced by PBPV. (A) The correlation between Ply-specific antibody titers and the OPA indices of the six serotypes at D30. **(B)** The correlation between the titers of antibody subclasses (IgG, IgM, IgG1, IgG2, IgG3 and IgG4) of three immunogens and the OPA indices for six serotypes at D30. Significance was measured using the Spearman correlation analysis in **A** and **B**. All p-values were adjusted for multiple testing using the Benjamini–Hochberg (BH) method. *p < 0.05, **p < 0.01, ***p < 0.001.

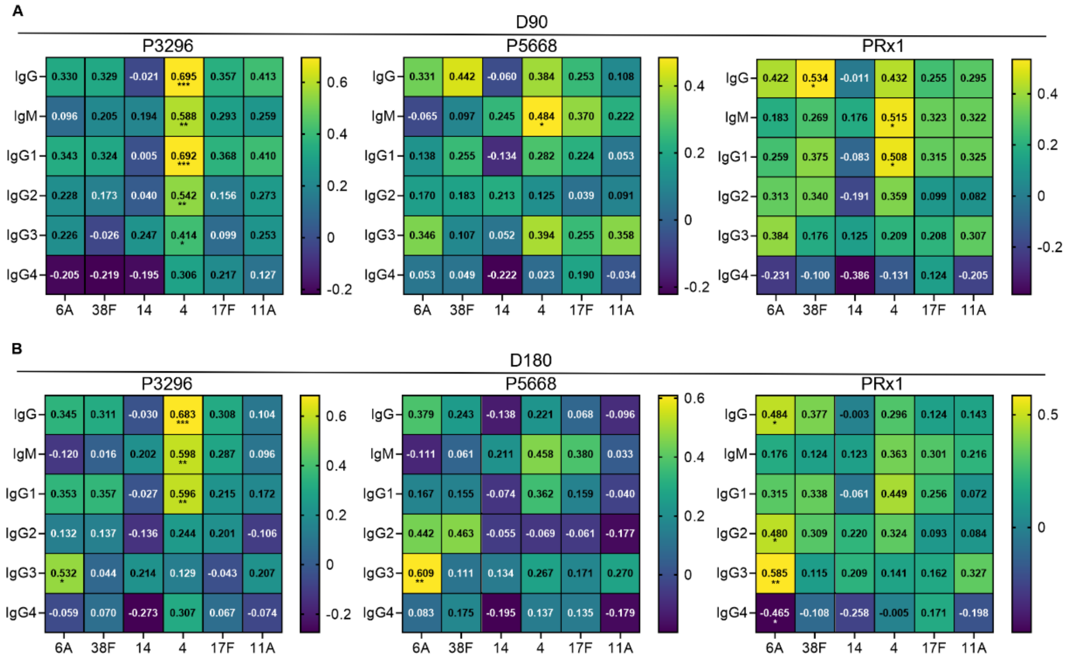

Supplemental Figure 8 Correlation between antibody titers and OPA indices at day 90 and 180 post-vaccination induced by PBPV. (A and **B)** The correlation between the titers of antibody subclasses of three PspA proteins and the OPA indices for six serotypes at D90 and D180. Significance was measured using the Spearman correlation analysis in **A** and **B**. All p-values were adjusted for multiple testing using the Benjamini–Hochberg (BH) method. $*p < 0.05$, $**p < 0.01$, $***p < 0.001$.

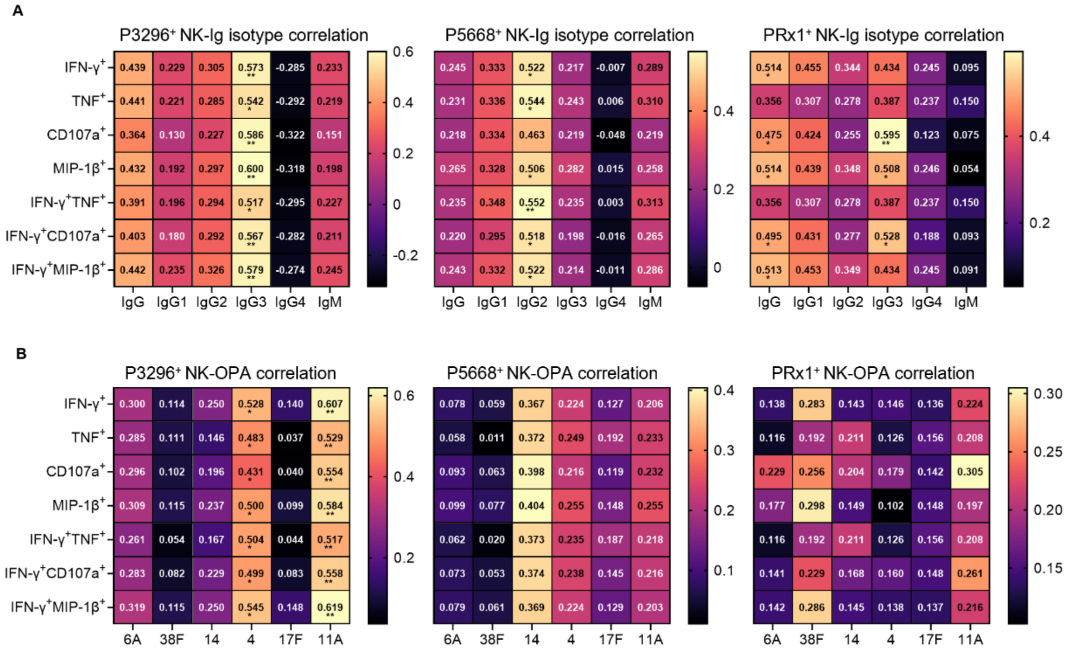

Supplemental Figure 9 Functional NK cell responses induced by PBPV. **(A)** Correlation analysis between the positive rate of cytokines secreted by NK cells activated by P3296, P5668, and PRx1-specific antibodies and the titers of corresponding different antibody subclasses. **(B)** Correlation analysis between the positive rate of cytokines secreted by NK cells activated by P3296, P5668, and PRx1-specific antibodies and the OPA indices of 6 serotypes. Significance was measured using the Spearman correlation analysis in **A** and **B**. All p-values were adjusted for multiple testing using the Benjamini–Hochberg (BH) method. $*p < 0.05$, $**p < 0.01$, $***p < 0.001$.
